# Mapping the landscape of managed entry agreements: a systematic review of global frameworks, system-level components, and implementation challenges

**DOI:** 10.3389/fphar.2026.1803870

**Published:** 2026-04-20

**Authors:** Hussain Abdulrahman Al-Omar, Asma Abdulaziz Almuhsin, Lolwa Hamad Almudaiyan, Amal Hassan Al-Najjar, Laila Carolina Abu Esba, Hind Almodaimegh, Jehan Alamre, Consuela Cheriece Yousef, Mansoor Ahmed Khan, Khalid AlYahya, Esraa S. Altawil, Abdualmajeed Saad Binjumayah, Faisal Awwadh Alharbi, Sultan Tawfiq AlShurbaji, Fatma Maraiki, Jaime Espín, Rosanna Tarricone, Panos Kanavos

**Affiliations:** 1 Department of Clinical Pharmacy, College of Pharmacy, King Saud University, Riyadh, Saudi Arabia; 2 General Directorate of Research and Studies, Deputyship of Planning and Institutional Excellence, Ministry of Health, Riyadh, Saudi Arabia; 3 Center for Health Technology Assessment, Ministry of Health, Riyadh, Saudi Arabia; 4 King Abdulaziz Medical City, Ministry of National Guard-Health Affairs, Ministry of National Guard, Riyadh, Saudi Arabia; 5 King Saud Bin Abdulaziz University for Health Sciences, Riyadh, Saudi Arabia; 6 King Abdullah International Medical Research Centre, Riyadh, Saudi Arabia; 7 Ministry of National Guard - Health Affairs, Riyadh, Saudi Arabia; 8 Logistics and Contracts Management, King Abdulaziz Medical City, Ministry of National Guard- Health Affairs, Ministry of National Guard, Riyadh, Saudi Arabia; 9 Imam Abdulrahman Bin Faisal Hospital, Ministry of National Guard, Health Affairs, Dammam, Saudi Arabia; 10 King Saud Bin Abdulaziz University for Health Sciences, Al Ahsa, Saudi Arabia; 11 King Abdullah International Medical Research Centre, Al Ahsa, Saudi Arabia; 12 King Abdulaziz Medical City, Ministry of National Guards Health Affairs, Jeddah, Saudi Arabia; 13 Department of Pharmaceutical Service, Prince Sultan Military Medical City, Riyadh, Saudi Arabia; 14 Corporate Pharmacy Service, King Saud University Medical City, Riyadh, Saudi Arabia; 15 Tawuniya Insurance Company, Riyadh, Saudi Arabia; 16 University of York, York, United Kingdom; 17 Pharmaceutical Care Division, King Faisal Specialist Hospital and Research Centre, Riyadh, Saudi Arabia; 18 Andalusian School of Public Health, Escuela Andaluza de Salud Pública (EASP), Granada, Spain; 19 CIBER en Epidemiología y Salud Pública (CIBERESP), CIBER of Epidemiology and Public Health (CIBERESP), Instituto de Investigación Biosanitaria ibs.GRANADA, Granada, Spain; 20 Department of Social and Political Sciences, Bocconi University, Milan, Italy; 21 Department of Health Policy, London School of Economics and Political Science, LSE Health-Medical Technology Research Group, London, United Kingdom

**Keywords:** challenges, framework, governance, health system, implementation, managed entry agreements, pharmaceutical policy, reimbursement

## Abstract

**Background:**

Managed Entry Agreements (MEAs) are pivotal for enabling access to innovative pharmaceuticals while mitigating financial risk and addressing evidence uncertainty. Despite conceptual acceptance, the essential requirements for their design and implementation are poorly documented, posing a challenge for evolving health systems. This study aimed to systematically review and map global evidence published in the literature on the existence and characterization of countries’ MEA policies, focusing on the core system-level components, governance, and operational frameworks for successful implementation, and their associated development and implementation challenges.

**Methods:**

A systematic review was conducted following PRISMA guidelines. MEDLINE and EMBASE were searched from inception to March 2025. Studies discussing governance, frameworks, legislation, or implementation of pharmaceutical MEAs were included. Data were extracted and synthesized narratively.

**Results:**

Of 96 included studies, most focused on European (43%) and North American (28%) systems, with a significant evidence gap for the Middle East and low-income countries. While situational analysis was the most common theme (63%), the literature predominantly catalogued barriers rather than providing operational solutions. Key barriers were inefficient regulatory frameworks (identified in 42% of studies), data infrastructure limitations (41%), and high administrative burden (38%). Stakeholder analysis highlighted underrepresentation of academia and civil society. A minority of studies (15%) focused on Advanced Therapy Medicinal Products (ATMPs).

**Conclusion:**

This review identifies a critical “how-to” gap in the MEA literature. While the value of MEAs is acknowledged, there is a stark deficit of actionable, system-level guidance on the regulatory, governance, and operational prerequisites for implementation. For health systems seeking to adopt MEAs, future efforts must shift from describing barriers to developing concrete implementation toolkits, legislative roadmaps, and fit-for-purpose payment models.

## 1 Introduction

Globally, health systems face mounting pressures driven by demographic shifts, including aging populations, the persistence of risk factors, and a growing burden of chronic diseases, all amid increasing financial constraints (Kankeu et al., 2013). Concurrently, rapid advances in health technologies—particularly pharmaceutical technologies—deliver substantial clinical benefits but at unprecedented cost. In Saudi Arabia, for instance, pharmaceutical expenditure is projected to increase from USD 11.9 billion in 2023 to USD 19.2 billion by 2030, representing one of the fastest growth rates in the region and accounting for a substantial share of total healthcare spending (GHE, 2024; BMI–A Fitch Solutions Company, 2024). This expansion is propelled by a shift toward innovative, curative, and transformative therapies targeting chronic diseases, genetic disorders, oncology, and other rare and complex conditions.

However, the adoption of these therapies entails substantially greater complexity than that of conventional pharmaceuticals (Hennessy et al., 2023). Advanced Therapy Medicinal Products (ATMPs), including gene and chimeric antigen receptor T cell therapies, encompass not only the therapeutic agent but also intricate associated services, such as specialized manufacturing, cryopreservation, and time-sensitive global supply chains. Furthermore, their administration requires certified sites of care, specialized laboratory infrastructure, and sophisticated logistical coordination to safeguard patient safety and treatment integrity (Cheema et al., 2025).

The proliferation of complex, curative, and transformative therapeutic modalities poses a significant challenge for health technology assessment (HTA) bodies and payers (Berishvili et al., 2024; Goncalves, 2025). These therapies frequently command prices that test the fiscal capacity of even the most affluent healthcare systems and are characterized by pervasive evidentiary uncertainty, particularly regarding long-term clinical outcomes and cost-effectiveness, thereby complicating their evaluation and reimbursement decision-making and creating critical access dilemma (Rudy, 2025; Zinzi et al., 2023).

Among the available pricing and reimbursement policy instruments, Managed Entry Agreements (MEAs) have emerged as a targeted mechanism for managing the inherent tension between access, uncertainty, and affordability associated with innovative therapies. MEAs are arrangements between manufacturers and payers or providers that enable access to coverage or reimbursement of a health technology subject to specific conditions (Wenzl and Chapman, 2019). They are broadly categorized into financial-based agreements (FBAs) or performance- or outcome-based agreements (PBAs/OBAs), and a third emerging type: service-based agreements (Dabbous et al., 2020). Although their design and intensity vary across jurisdictions, MEAs are instrumental for payers in managing financial risk while maintaining patients’ access to innovative therapies (Alum et al., 2025).

For evolving or transitional health systems, understanding the practical requirements for implementing and operationalizing MEAs is crucial. Such systems are frequently confronted with the dual imperative of concurrently developing HTA capabilities and capacities while managing the complexities of introducing novel reimbursement mechanisms, a context in which MEAs remain in their formative stages as a policy instrument. Within the Saudi health system, which is undergoing a major health transformation, including the establishment of a center for health technology assessment, MEAs remain a relatively underdeveloped and infrequently used policy tool (Al-Omar et al., 2020a; Al-Omar et al., 2020b). A 2020 study on MEAs in Saudi Arabia indicated growing stakeholder interest in their adoption to balance patient access, accelerate therapy uptake, and support financial sustainability, while noting that FBAs predominated due to their relative simplicity (Al-Omar et al., 2021).

Despite a growing body of literature on MEAs, no comprehensive review has described countries MEAs in relation to their essential system-level core components and prerequisites for implementation and operationalization. This includes regulatory and operational frameworks, enabling legislation, explicit product eligibility criteria, and robust governance structures. This study aimed to systematically review and map global evidence published in the literature on the existence and characterization of countries’ MEA policies, focusing on the core system-level components, governance, and operational frameworks for successful implementation, and their associated development and implementation challenges. The objectives were to explore existing global MEA frameworks; identify foundational governance and operational prerequisites; map pivotal stakeholders involved; and characterize implementation challenges. The evidence generated by this review may inform the development of a structured MEA framework and governance model to support strategic and sustainable MEA implementation across different national contexts, with particular relevance for the evolving health policy landscape in Saudi Arabia and potentially the wider region.

## 2 Methods

### 2.1 Study design and reporting

We conducted a systematic literature review guided by the study aims and objectives. The review followed the Preferred Reporting Items for Systematic Reviews and Meta-Analyses (PRISMA) guidelines to ensure methodological rigor and transparency (Page et al., 2021).

### 2.2 Data sources and search strategy

We executed a systematic literature search across two major electronic databases: the National Library of Medicine (MEDLINE) and the Excerpta Medica Database (EMBASE). We conducted the initial search in March 2024, with updates in November 2024 and a final update in March 2025. The search included studies published in peer-reviewed journals from database inception through March 2025.

The search strategy was developed iteratively, beginning with a preliminary non-structured search to identify key terms that informed the final structured strategy (Supplementary Table S1). Core search terms included “managed entry agreement,” “framework,” “requirements,” “health technology assessment,” “innovative payment model,” and “access.” To maximize sensitivity, the strategy combined free-text keywords, Medical Subject Headings (MeSH), and EMTREE terms, alongside truncation symbols to capture word stems and Boolean operators (AND, OR, NOT) to link terms. The search strategy and associated terms were developed in consultation with subject matter experts (co-authors) and formally reviewed by an information specialist.

### 2.3 Study selection and eligibility criteria

#### 2.3.1 Studies were included if they met the following predefined criteria:

- Focused on any type of MEA for pharmaceutical products.
- Discussed one or more of the following topics: governance, policies, legislation, regulations, frameworks, barriers, challenges, implementation processes, or impact assessment.
- Published in English language.

We excluded:

- Studies addressing payment agreements for non-pharmaceutical healthcare services.
- Studies focused on medical devices or diagnostics without a primary pharmaceutical focus.
- Theoretical frameworks without official implementation.
- Studies that do not present independent primary data but instead derive their findings exclusively from an already-included original study.
- Non-English publications.
- Systematic literature reviews.

### 2.4 Screening, data extraction, and synthesis

We imported all identified citations into EndNote® version 20 (Thomson Reuters, Philadelphia, United States of America). Following duplicate removal, we conducted a multi-stage screening process. Four independent reviewers screened titles and abstracts against eligibility criteria. The same reviewers retrieved and assessed full texts of potentially relevant records. Discrepancies at any stage were resolved through discussion or, if needed, consultation with a fifth reviewer (the lead author). The reference lists of all included records were also hand-searched for additional relevant studies.

Three reviewers extracted data using a standardized, piloted form, and two senior reviewers verified the extracted information. Extracted data encompassed bibliographic information (authors, publication year, country); study objectives; healthcare system context; involved stakeholders; MEA type (financial or performance-based); technology type, including identification of ATMPs; responsible entity; details of the MEA framework; nature of product uncertainties; funding modalities; and reported advantages, disadvantages, challenges, and risks.

We organized the extracted data in a standardized Microsoft Excel file. Given the heterogeneity across study designs and outcomes, we performed a narrative, descriptive synthesis supported by descriptive statistical analyses of frequencies. The findings are presented in summary tables aligned with study objectives.

### 2.5 Risk of bias assessment

We assessed methodological quality using the Critical Appraisal Skills Programme (CASP) checklist (Critical Appraisal Skills Programme, 2020). Reviewers conducted quality appraisals independently and resolved disagreements through consensus discussion. Risk assessment and confidence in the body of evidence tables of the included studies is provided in Supplementary Table S2, S3.

## 3 Results

### 3.1 Study selection

**FIGURE 1:**
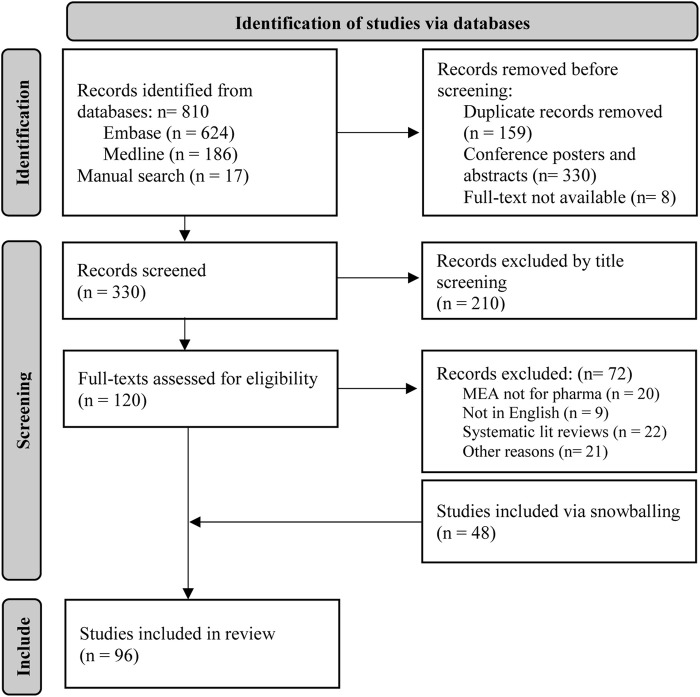
PRISMA diagram of the literature search and inclusion of publications.

The systematic search across MEDLINE and EMBASE identified 810 records. After removal of 159 duplicates, 330 conference abstracts, and 8 records without full-text access, 330 records underwent title and abstract screening. We excluded 210 records as irrelevant to the study objectives. The remaining 120 full-text records underwent comprehensive eligibility assessment. Following systematic evaluation and supplemental hand-searching of reference lists, 96 records were ultimately determined to satisfy the inclusion criteria and were included in the final analysis. Figure 1 presents the study selection process using a PRISMA flow diagram. Table 1 Presents all included studies in the systematic review while Supplementary Table S4 provides a full list of excluded records with reasons for exclusion.

**TABLE 1:** Overview of publications discussing MEA framework, governance, legislation, policies, and regulations.

#	Author (Year)	Country	Targeted sector (Public/Private/Both)	Stakeholders involved in the study	Theme (One or more)	MEA (OBA/FBA/Both)	ATMPs included (Yes/No)
1	Bartos (2024)	France	Public	In; C; SPs; HTA; Rg; PM; S&A; Pt; M	F; G; L; R; P; SA	OBA	Yes
2	Bhuiyan et al. (2024)	United States of America	Both	In; Pr; SPs; Rg; A; M	P; R; SA; I	OBA	Yes
3	Callenbach et al. (2024)	United States of America; United Kingdom; Netherlands	Public	Pr; HTA; SPs; Rg; (NGO/HTA); HTA; Pt; M	F; I; R	OBA	Yes
4	Dayer et al. (2024)	Not mentioned	N/A	Pr; HTA; SPs; Rg; Pt; M	F; G; R; I; SA	OBA	Yes
5	Hospod et al. (2024)	Czech	Both	In; Rg; Pt; SPs; M; HCPs	F; G; L; I; SA	OBA	No
6	Towse and Fenwick (2024)	United States of America, Europe	Public	Pr; HTA; Pt; M	G; R; P	OBA	No
7	Trigg et al. (2024)	United Kingdom	Public	M; Pt; HTA; Rg; SPs; Pr; C	F; SA	Both	No
8	Kim et al. (2023)	Korea	Public	HTA; In; Rg; M	F; IA; P; SA	Both	No
9	McPhail and Bubela (2023)	Canada	Both	HTA; Rg; In; M; HCPs; Pt	F; G; L; R; P; I; SA	Both	No
10	Meresz and Gaal (2023)	Hungary	Public	In; Rg; HTA; Pr; AP; M	G; L; P	Both	No
11	Neumann et al. (2023)	United States of America	Public	Rg; Pr; M; HTA; Pt; PM; RD; C	G; L; R; P	Both	Yes
12	Quinn et al. (2023a)	United States of America	Public	Pr; M; In; Pt; PM; S&A	P; R; IA; SA	OBA	Yes
13	Quinn et al. (2023b)	United States of America	Public	Pr; In; S&A; M; Pt	R; P; SA	OBA	No
14	Cheung et al. (2022)	Canada	Both	Pr; M; In; Rg; HTA; Pt; HCPs	F; I	OBA	No
15	Eichler et al. (2022)	Other	Both	Pr; In; SPs; Rg; Pt; M; HCPs	F; G; R; I; SA	Both	Yes
16	Russo (2022)	Italy	Public	SPs; Rg; Pr; Pt; M	F; SA; IA	Both	No
17	Sandhu et al. (2022)	United States of America	Private	Pr; In; Rg; Pt; M	F; SA; IA	OBA	No
18	Simoens et al. (2022)	Europe	Public	SPs; Pr; Rg; HTA; (NGO/HTA); Pt; M	F; G; R; P; SA	Both	Yes
19	Stafinski et al. (2022)	Australia; Canada; Catalonia; France; Germany; Italy; Spain; Sweden; Netherlands; New Zealand; United Kingdom	Both	Pr; Pt; SPs; PM; AP; HTA; Rg; A; M	F; G; R; P; SA; I	Both	No
20	Xoxi et al. (2022)	Italy	Public	Rg; SPs; C; S&A; Pt; RD; Pr; M; HCPs	F; L; R; P; IA; I; SA	OBA	Yes
21	Al-Omar et al. (2021)	Saudi Arabia	Public	M, HTA, Pr, SPs, A, C	F; L; R; G; P; I; SA	Both	Yes
22	Bang and Lee (2021)	South Korea	Public	In; Rg; HCPs; Pts; M	L; R; P; I; SA	OBA	No
23	Kim et al. (2021)	South Korea	Public	In; HTA; Rg; Pt; M	SA; IA	Both	No
24	Pereira et al. (2021)	Portugal	Both	SPs; Rg; S&A; A; Pt; C HTA; M; Pr	F; SA; I	Both	Yes
25	Gamba et al. (2020)	Belgium; Italy; Netherlands; England	Public	N/A	F	Both	No
26	Kannarkat et al. (2020)	United States of America; United Kingdom	Both	In; Pr; SPs; Pt; M	F; IA	OBA	No
27	Lopez et al. (2020)	United States of America	Both	In; Pt; Pr; C; SPs; Rg; (NGO/HTA); HTA; M	F; P; R; I; IA	Both	Yes
28	Goodman et al. (2019)	United States of America	Both	In; C; Pr; Rg; SPs; M; Pt	F; R; P; I; SA	OBA	No
29	Holleman et al. (2019)	Netherlands	Public	Pr; SPs; Pt; M	P; IA	Both	No
30	Lorente et al. (2019)	Spain	Public	Rg; SPs; Pt; M; HCPs	I; SA	Both	No
31	Makad et al. (2019)	Netherlands	Public	HTA; M; Pr; In; S&A; Pt; SPs	G; P; SA; IA	Both	No
32	Mundy et al. (2019)	Asia: Indonesia; India; Korea; Malaysia; the Philippines; Singapore; Taiwan; Thailand; Vietnam	Both	M; In; HTA; Pr; Pt; HCPs	G; I	Both	No
33	Triki et al. (2019)	Others	Public	Rg; In; C; M	SA	FBA	No
34	Wenzl and Chapman (2019)	OECD countries and Europe	Public	N/A	F	Both	No
35	Yoo et al. (2019)	South Korea	Public	Rg; In; HTA; C; Pt; M	R; P; SA; IA	FBA	No
36	Bouvy et al. (2018)	Europe	Public	Pr; Rg; C; HTA; Pt; SPs; A; M	F; I; R	both	No
37	Brow et al. (2018)	United States of America	Both	Pr; M; In; Pt	F; G; IA	OBA	No
38	Duhig et al. (2018)	United States of America	Both	In; SPs; Pr; Rg; M; HCPs	R; SA; I; IA	OBA	No
39	Dunlop et al. (2018)	France; Germany; Italy; Spain; United Kingdom	Both	Pr; In; SPs; HTA; Pt; M; HCPs	P; SA; I	Both	No
40	Goncalves et al. (2018)	Portugal	Public	SPs; Rg; HTA; Pt; M; Pr; HCPs	L; R; I; SA; IA	Both	No
41	Kefalas et al. (2018)	United Kingdom	Public	Pr; PM; HTA; Rg; SPs; Pt; M; HCPs	IA	OBA	Yes
42	Mahjoub et al. (2018)	Canada	Public	N/A	F	OBA	No
43	Maskineh and Nasser (2018)	Middle East/North Africa (MENA)	Public	Pr; A; HTA; In; Pt; HCPs; M	SA; G; I	Both	No
44	Robinson et al. (2018)	Australia	Public	PM; C; HCPs; HTA; M; Pr	I; P; SA	Both	No
45	Tuffaha and Scuffham (2018)	Australia	Public	AP; PM; SPs; HTA; Rg; Pt; M; HCPs	G; P; SA; I	FBA	No
46	Calabrese et al. (2017)	United States of America	Both	Pr; M; SPs; Rg; Pt; HCPs	F; I; G; SA	OBA	No
47	Carlson et al. (2017)	United States of America; United Kingdom; Sweden; Italy; Netherlands; Australia	Public	Pr; HTA; Rg; RD; In; Pt; M	SA; I	Both	No
48	Clopes et al. (2017)	Spain	Public	SPs; Pt; M; Pr	IA	OBA	No
49	Goble et al. (2017)	United States of America	Both	C; In; Pr; Pt; M	F; I; SA	OBA	No
50	Grimm et al. (2017)	United Kingdom	Public	HTA; Pr; Pt; M	F	Both	No
51	Jorgensen and Kefalas (2017)	England	both	SPs; HTA; Rg; A; Pt; M; Pr	IA; L	OBA	Yes
52	Kanavos et al. (2017)	United Kingdom	Both	Pr; In; SPs; Rg; HTA; Pt; M	F; G; SA; IA	Both	No
53	Kim et al. (2017)	South Korea	Public	HTA; In; Rg; C; Pt; M	R; P; SA	Both	No
54	Nazareth et al. (2017)	United States of America; Europe	Both	Pr; In; C; Rg; HTA; PM; M	SA; I	OBA	No
55	Pauwels et al. (2017)	Europe (Belgium, Netherlands, Scotland, England and Wales, Sweden, Italy, Czech Republic and France)	Public	Pr; In; Rg; HCPs; HTA; Pt; M	R; SA	Both	No
56	Seeley and Kesselheim (2017)	United States of America	Both	In; Pr; C; Rg; Pt; M; SPs; HCPs	IA	OBA	No
57	Toumi et al. (2017)	France	Both	Pr; M; HTA; Pt	F	OBA	No
58	Yeung et al. (2017)	United States of America	Public	In; HCPs; Rg; Pt; M	G; L; P; SA; I	Both	No
59	Claxton et al. (2016)	United Kingdom	Public	N/A	F	Both	No
60	Faulkner et al. (2016)	EU; United States of America	Both	SPs; PM; Pr; Rg; In; HTA; Pr/AP; M	F; G; I; SA; R; P; IA	Both	No
61	Thompson et al. (2016)	Canada	Both	Pt; M; Pr; Rg; SPs; RD; HTA; C; HCPs	G; SA; IA; I	Both	No
62	Drummond (2015)	United Kingdom	Public	Pr; SPs; M; HCPs	F; I	OBA	No
63	Ferrario et al. (2015)	Belgium; England; Netherlands; Sweden	Public	Pr; PM; SPs; HTA; In; Pt; M	F; G; P; SA; I	Both	No
64	Garattini et al. (2015)	Italy	Public	Rg; SPs; Pt; M; Pr	SA; IA	Both	No
65	Garrison et al. (2015)	United States of America	Both	Pr; C; In; Pt; M	P; SA; I	Both	No
66	Lewis et al. (2015)	Australia	Both	Pr; HTA; SPs; In; Rg; C; Pt; M; HCPs	F; G; R	OBA	No
67	Lu et al. (2015)	United States of America	Public	Pr; In; HTA; C; Rg; Pt; HCPs; M	SA	Both	No
68	Navarria et al. (2015)	Italy	Public	SPs; Rg; C; M; Pt; Pr	G; SA	Both	No
69	Carlson et al. (2014)	United States of America	Both	SPs; Pr; HTA; Rg; C; Pt; M; HCPs	SA	Both	No
70	Gibson and Lemmens (2014)	United Kingdom	Public	Pr; SPs; Rg; HTA; Pt; M; HCPs	G; L; R; P; SA	OBA	No
71	Launois et al. (2014)	France	Public	In; Pt; M	F; P; I	OBA	No
72	Eckermann and Willan (2013)	Canada	Public	N/A	F	OBA	No
73	Garrison et al. (2013)	United Kingdom; United States of America; France; Italy; Netherlands	Both	In; Pr; SPs; Rg; RD; C; PM; HTA; M; HCPs	F; G; I; R; P	Both	No
74	Morel et al. (2013)	Europe (Belgium, England and Wales, France, Germany, Italy, Netherlands and Sweden)	Both	In; Rg; HTA; C; Pr; SPs; M; PM; Pt; HCPs	F; P; R; SA	Both	No
75	Evans (2012)	United Kingdom	NA	N/A	F	N/A	No
76	Walker et al. (2012)	United Kingdom	Public	N/A	F	Both	No
77	Wonder et al. (2012)	Australia	Both	HTA; Rg; SPs; Pt; S&A; M; Pr	F; G; R; P; SA; IA	Both	No
78	Annemans et al. (2011)	EU	Public	Pr; Rg; HTA; S&A; Pt; M	F; G; P; SA	Both	No
79	Barros (2011)	N/A	Public	N/A	F	OBA	No
80	Garattini and Casadei (2011)	Italy	Public	SPs; Rg; Pt; M; HCPs; Pr	SA; I; P	Both	No
81	Jaroslawski and Toumi (2011a)	Europe (United Kingdom, France, Italy, Sweden and Denmark)	Both	Pr; C; Rg; HTA; PM; In; SPs; M	F; P; SA	Both	No
82	Jaroslawski and Toumi (2011b)	United Kingdom	Public	PM; SPs; HTA; M; Rg; Pr	F	Both	No
83	Klemp et al. (2011)	Europe; Australia; Canada	Both	In; HTA; SPs; Pt; M	F; G; P; I	Both	No
84	Menon et al. (2011)	Canada	Public	Pt; SPs; Rg; HTA; C; M; HCPs; Pr	F; G; P; I	Both	No
85	Neumann et al. (2011)	United States of America	Both	Pr; SPs; In; HTA; Pt; M	G; I; SA	OBA	No
86	Adamski et al. (2010)	Europe (Belgium, Estonia, Germany, Sweden, Denmark, France, Hungary, Italy, Lithuania, Portugal), Australia, United Kingdom, Canada	Both	In; SPs; HTA; Rg; Pt; M; HCPs	F; P; SA	Both	No
87	Carlson et al. (2010)	United Kingdom; Sweden; United States of America; France; Australia	Both	Pr; SPs; In; Rg; S&A; Pt; HTA; M	SA; IA; P	OBA	No
88	McCabe et al. (2010)	United Kingdom	Both	PM; M; Pr; Pt; HCPs; HTA; RD	F; G; I; SA	OBA	No
89	Menon et al. (2010)	Alberta, Canada	Both	SPs; Pt; M; HTA; HCPs; Pr	F; G; I	Both	No
90	Towse and Garrison (2010)	France; Australia; New Zealand; United Kingdom; United States of America	Both	Pr; M; Pt	F; P; IA	Both	No
91	Trueman et al. (2010)	Canada	Both	Ins; SPs; Pr; HTA; Rg; Pt; M	F; G; SA; IA	OBA	No
92	Willis et al. (2010)	Sweden	Public	HTA; Rg; Pt; M	F; SA	OBA	No
93	Carlson et al. (2009)	United States of America	Both	In; Pr; S&A; SPs; Rg; Pt; RD; M; HCPs	F; P; SA; I	OBA	No
94	Williamson and Thomson (2009)	United Kingdom	Public	Rg; PM; SPs; HTA; Pt; M; HCPs; Pr	IA; SA	Both	No
95	Hutton et al. (2007)	United Kingdom	Public	Pr; HTA; SPs; Pt; M	F; G; P; R; I; IA	OBA	No
96	Segal and Whinston (2002)	United States of America	N/A	N/A	F	N/A	No

Stakeholders: (Pr, Payer; HTA, health technology assessment; P, Patient & advocacy; M, Manufacturer & developer; In, Insurance; Rg, Regulator (Council representative; early access); SPs, Service providers; A, academia; C, Committees (formulary management; PTC; Utilization review sub-com; evaluation; appraisal; Advisory); AP, access program; PM, policymaker; S&A, societies and associations; RD, Research and data).

Themes: (F, framework; L, legislation; R, regulation; G, governance; P, policies; I, implementation; SA, situational analysis; IA, impact assessment). UK , united kingdom; USA , United States of America. N/A, not available.

### 3.2 Characteristics of included publications

**FIGURE 2:**
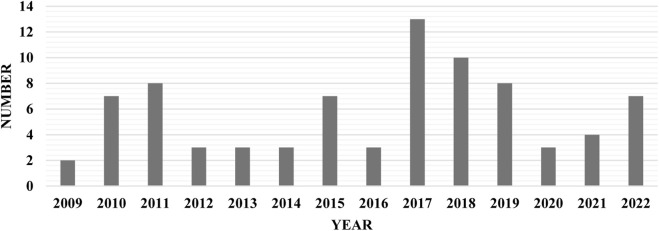
Number of MEAs publications over years.

Temporal analysis revealed a marked increase in scholarly attention to MEAs over time, progressing from sporadic publications in the early 2000s to a peak of 13 publications in 2017. Publications from 2017 to 2024 accounted for 60% (n = 58) of included studies, reflecting heightened academic and policy interest aligned with the emergence of high-cost innovative, curative and transformative therapies (Figure 2).

**FIGURE 3:**
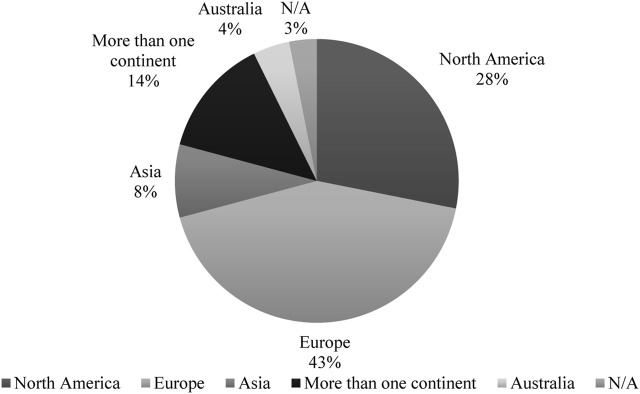
Percentage of MEAs publications across continents.

Geographically, European health systems were most frequently studied (43%), followed by North American systems (28%). Asian countries appeared in 8% of publications, while multicontinental comparative analyses constituted 14% of the corpus. Only 2% of studies focused on Middle Eastern/North African (MENA) regions and none addressed low-income countries, highlighting substantial geographical disparities in the evidence base (Figure 3).

### 3.3 Targeted sectors

Analysis of targeted healthcare sectors revealed a predominant focus on public health systems. Of the 96 included studies, 53 (55%) examined MEA implementation exclusively in public systems. Studies addressing both public and private sectors accounted for 39 publications (40%), while only one study (1.1%) focused solely on the private sector. Three studies (3.1%) did not specify the sector. This distribution indicates that current MEA evidence is primarily derived from publicly funded or mandated health systems.

### 3.4 Thematic focus of studies

Systematic coding of primary themes demonstrated that situational analysis was the most frequently discussed topic, appearing in 60 studies (62.5%), followed by frameworks (56 studies, 58.3%). This pattern underscores a strong emphasis on contextual assessment and baseline structural conditions for MEAs. Publications related to implementation, system-level policy, governance, regulation, impact assessment, and legislation were comparatively limited. Substantial thematic co-occurrence emerged across conceptually linked domains. Implementation and situational analysis co-occurred most frequently, appearing together in 31 studies (32.3%), suggesting that contextual understanding underpins deployment and implementation. Similarly, policy and situational analysis also showed notable co-occurrence, reinforcing their interdependence in reimbursement decision-making architectures. The co-occurrence of frameworks and implementation (27.0%) further emphasizes the intrinsic link between structural design and operational execution in MEA discourse. Although all studies provided country-specific details at varying levels of depth, none comprehensively addressed system-level requirements across the full policy cycle, nor offered transferable guidance to inform MEA development, deployment and and/or implementation across health systems.

### 3.5 Stakeholder engagement

Analysis of stakeholder representation and engagement revealed that more than half of the included studies examined MEAs in the context of publicly funded health systems, compared with private or insurance-based systems. Manufacturers (91%, n = 88), patient and advocacy groups (77%, n = 74), and payers (74%, n = 71) were the most frequently cited stakeholders, reflecting their central roles in MEA design and implementation. Regulators (63.5%, n = 61), HTA bodies (60%, n = 58), and healthcare providers (57.3%, n = 55) comprised the next tier of frequently engaged stakeholders. Notable gaps in stakeholder representation emerged for scientific societies and associations (10.4%, n = 10), academia (7.3%, n = 7), and research and data organizations (7.3%, n = 7), suggesting limited systematic engagement of these crucial stakeholder groups in MEA discourse despite their significant vested interests in therapeutic access and outcomes.

### 3.6 ATMPs inclusion

Analysis of technology focus revealed that 14 of the 96 included publications (14.6%) specifically addressed ATMPs, whereas the majority (85.4%) discussed MEAs in the context of traditional pharmaceuticals. This distribution indicates that although ATMPs represent a specialized and growing area of policy concern, they remain a minority focus within the broader MEA literature. The substantial subset of ATMP-focused literature nonetheless highlights the distinctive policy challenges posed by curative, high-cost therapies when assessed within reimbursement frameworks originally designed for chronic, small-molecule drugs. This body of literature highlights the unique evidentiary, financial, and operational considerations required for these therapies.

### 3.7 Barriers and challenges for MEA implementation

Analysis of the literature revealed that barriers and challenges for MEA implementation were extensively documented. The systematic examination identified 32 discrete barriers and challenges grouped into eleven thematic categories, derived from 68 publications included in this review.

### 3.8 Barriers and challenges categorization

A comprehensive analysis substantial barriers revealed across several critical domains that impede effective MEA implementation (Table 2).

**TABLE 2:** MEAs implementation challenges and barriers across countries.

Theme	(n) References	Subtheme	(n) References
Regulatory framework	**(40)** (Al-Omar et al., 2021); (Klemp et al., 2011); (Bartos, 2024); (Bhuiyan et al., 2024); (Hospod et al., 2024); (Kim et al., 2023); (McPhail and Bubela, 2023); (Meresz and Gaal, 2023); (Neumann et al., 2023); (Quinn et al., 2023b); (Eichler et al., 2022); (Simoens et al., 2022); (Xoxi et al., 2022); (Lopez et al., 2020); (Goodman et al., 2019); (Lorente et al., 2019); (Yoo et al., 2019); (Bouvy et al., 2018); (Duhig et al., 2018); (Goncalves et al., 2018); (Maskineh and Nasser, 2018); (Robinson et al., 2018); (Tuffaha and Scuffham, 2018); (Calabrese et al., 2017); (Clopes et al., 2017); (Goble et al., 2017); (Kanavos et al., 2017); (Kim et al., 2017); (Nazareth et al., 2017); (Yeung et al., 2017); (Thompson et al., 2016); (Garrison et al., 2015); (Lu et al., 2015); (Carlson et al., 2014); (Gibson and Lemmens, 2014); (Garrison et al., 2013); (Adamski et al., 2010); (Menon et al., 2010); (Towse and Garrison, 2010); (Hutton et al., 2007)	Inefficient regulatory framework (unclear/lack)	**(18)** (Al-Omar et al., 2021); (Hospod et al., 2024); (Meresz and Gaal, 2023); (Neumann et al., 2023); (Quinn et al., 2023b); (Eichler et al., 2022); (Yoo et al., 2019); (Duhig et al., 2018); (Robinson et al., 2018); (Clopes et al., 2017); (Goble et al., 2017); (Kanavos et al., 2017); (Nazareth et al., 2017); (Thompson et al., 2016); (Carlson et al., 2014); (Garrison et al., 2013); (Klemp et al., 2011); (Towse and Garrison, 2010)
		Regulations need to be updated/evolved	**(10)** (Al-Omar et al., 2021); (Bartos, 2024); (Bhuiyan et al., 2024); (Meresz and Gaal, 2023); (Lopez et al., 2020); (Lorente et al., 2019); (Bouvy et al., 2018); (Goncalves et al., 2018); (Kim et al., 2017); (Yeung et al., 2017)
		Lack of exit strategy regulations	**(10)** (Al-Omar et al., 2021); (Kim et al., 2023); (McPhail and Bubela, 2023); (Xoxi et al., 2022); (Robinson et al., 2018); (Tuffaha and Scuffham, 2018); (Lu et al., 2015); (Gibson and Lemmens, 2014); (Adamski et al., 2010); (Hutton et al., 2007)
		Lack of governance structure	**(7)** (Al-Omar et al., 2021); (Hospod et al., 2024); (Simoens et al., 2022); (Gibson and Lemmens, 2014); (Garrison et al., 2013); (Adamski et al., 2010); (Menon et al., 2010)
		Fragmented healthcare systems (lack of connection between health system structure and the framework)	**(6)** (Al-Omar et al., 2021); (McPhail and Bubela, 2023); (Maskineh and Nasser, 2018); (Calabrese et al., 2017); (Nazareth et al., 2017); (Garrison et al., 2015)
		Poor regulatory-industry early dialogue	**(4)** (Al-Omar et al., 2021); (Lopez et al., 2020); (Goodman et al., 2019); (Duhig et al., 2018)
Data resources and infrastructure	**(39)** (Al-Omar et al., 2021); (Klemp et al., 2011); (Bhuiyan et al., 2024); (Dayer et al., 2024); (Hospod et al., 2024); (Kim et al., 2023); (McPhail and Bubela, 2023); (Quinn et al., 2023b); (Cheung et al., 2022); (Eichler et al., 2022); (Simoens et al., 2022); (Stafinski et al., 2022); (Xoxi et al., 2022); (Kannarkat et al., 2020); (Goodman et al., 2019); (Lorente et al., 2019); (Makad et al., 2019); (Bouvy et al., 2018); (Duhig et al., 2018); (Goncalves et al., 2018); (Maskineh and Nasser, 2018); (Robinson et al., 2018); (Tuffaha and Scuffham, 2018); (Carlson et al., 2017); (Clopes et al., 2017); (Nazareth et al., 2017); (Pauwels et al., 2017); (Yeung et al., 2017); (Faulkner et al., 2016); (Thompson et al., 2016); (Garrison et al., 2015); (Lu et al., 2015); (Navarria et al., 2015); (Gibson and Lemmens, 2014); (Annemans et al., 2011); (Neumann et al., 2011); (Carlson et al., 2010); (Towse and Garrison, 2010); (Carlson et al., 2009)	Data quality	**(17)** (Al-Omar et al., 2021); (Bhuiyan et al., 2024); (Dayer et al., 2024); (Hospod et al., 2024); (Kim et al., 2023); (McPhail and Bubela, 2023); (Cheung et al., 2022); (Xoxi et al., 2022); (Kannarkat et al., 2020); (Bouvy et al., 2018); (Duhig et al., 2018); (Robinson et al., 2018); (Tuffaha and Scuffham, 2018); (Faulkner et al., 2016); (Navarria et al., 2015); (Annemans et al., 2011); (Neumann et al., 2011)
		Lack of data infrastructure	**(14)** (Al-Omar et al., 2021); (Quinn et al., 2023b); (Cheung et al., 2022); (Eichler et al., 2022); (Simoens et al., 2022); (Stafinski et al., 2022); (Makad et al., 2019); (Bouvy et al., 2018); (Robinson et al., 2018); (Carlson et al., 2017); (Pauwels et al., 2017); (Faulkner et al., 2016); (Lu et al., 2015); (Gibson and Lemmens, 2014)
		Information system limitations	**(13)** (Al-Omar et al., 2021); (Bhuiyan et al., 2024); (McPhail and Bubela, 2023); (Goodman et al., 2019); (Goncalves et al., 2018); (Maskineh and Nasser, 2018); (Clopes et al., 2017); (Nazareth et al., 2017); (Thompson et al., 2016); (Garrison et al., 2015); (Neumann et al., 2011); (Carlson et al., 2010); (Towse and Garrison, 2010)
		Lack or limited registries	**(8)** (Al-Omar et al., 2021); (McPhail and Bubela, 2023); (Quinn et al., 2023b); (Xoxi et al., 2022); (Maskineh and Nasser, 2018); (Garrison et al., 2015); (Klemp et al., 2011); (Neumann et al., 2011)
​	​	Monitoring and follow up system limitations	**(7)** (Al-Omar et al., 2021); (Lorente et al., 2019); (Clopes et al., 2017); (Yeung et al., 2017); (Garrison et al., 2015); (Lu et al., 2015); (Carlson et al., 2009)
Administrative burden	**(36)** (Al-Omar et al., 2021); (Klemp et al., 2011); (Dayer et al., 2024); (McPhail and Bubela, 2023); (Meresz and Gaal, 2023); (Eichler et al., 2022); (Sandhu et al., 2022); (Simoens et al., 2022); (Pereira et al., 2021); (Kannarkat et al., 2020); (Goodman et al., 2019); (Holleman et al., 2019); (Lorente et al., 2019); (Mundy et al., 2019); (Bouvy et al., 2018); (Goncalves et al., 2018); (Maskineh and Nasser, 2018); (Tuffaha and Scuffham, 2018); (Jorgensen and Kefalas, 2017); (Nazareth et al., 2017); (Pauwels et al., 2017); (Toumi et al., 2017); (Yeung et al., 2017); (Faulkner et al., 2016); (Thompson et al., 2016); (Ferrario et al., 2015); (Garrison et al., 2015); (Lu et al., 2015); (Carlson et al., 2014); (Garrison et al., 2013); (Annemans et al., 2011); (Neumann et al., 2011); (Adamski et al., 2010); (Carlson et al., 2010); (Carlson et al., 2009); (Hutton et al., 2007)	High administrative burden	**(34)** (Al-Omar et al., 2021); (Dayer et al., 2024); (McPhail and Bubela, 2023); (Meresz and Gaal, 2023); (Eichler et al., 2022); (Sandhu et al., 2022); (Simoens et al., 2022); (Pereira et al., 2021); (Kannarkat et al., 2020); (Goodman et al., 2019); (Holleman et al., 2019); (Lorente et al., 2019); (Mundy et al., 2019); (Bouvy et al., 2018); (Goncalves et al., 2018); (Maskineh and Nasser, 2018); (Jorgensen and Kefalas, 2017); (Nazareth et al., 2017); (Pauwels et al., 2017); (Toumi et al., 2017); (Yeung et al., 2017); (Faulkner et al., 2016); (Thompson et al., 2016); (Ferrario et al., 2015); (Garrison et al., 2015); (Lu et al., 2015); (Carlson et al., 2014); (Garrison et al., 2013); (Annemans et al., 2011); (Klemp et al., 2011); (Neumann et al., 2011); (Adamski et al., 2010); (Carlson et al., 2010); (Carlson et al., 2009)
		Reporting and monitoring burden	**(11)** (Al-Omar et al., 2021); (Eichler et al., 2022); (Bouvy et al., 2018); (Goncalves et al., 2018); (Tuffaha and Scuffham, 2018); (Yeung et al., 2017); (Lu et al., 2015); (Garrison et al., 2013); (Annemans et al., 2011); (Adamski et al., 2010); (Hutton et al., 2007)
Complexity	**(27)** (Al-Omar et al., 2021); (Klemp et al., 2011); (Bartos, 2024); (Bhuiyan et al., 2024); (McPhail and Bubela, 2023); (Quinn et al., 2023a); (Quinn et al., 2023b); (Eichler et al., 2022); (Stafinski et al., 2022); (Pereira et al., 2021); (Lorente et al., 2019); (Mundy et al., 2019); (Bouvy et al., 2018); (Goncalves et al., 2018); (Maskineh and Nasser, 2018); (Tuffaha and Scuffham, 2018); (Goble et al., 2017); (Nazareth et al., 2017); (Seeley and Kesselheim, 2017); (Faulkner et al., 2016); (Drummond, 2015); (Garrison et al., 2015); (Lu et al., 2015); (Navarria et al., 2015); (Annemans et al., 2011); (Neumann et al., 2011); (Hutton et al., 2007)	Implementation complexity	**(22)** (Al-Omar et al., 2021); (Bartos, 2024); (Bhuiyan et al., 2024); (McPhail and Bubela, 2023); (Quinn et al., 2023a); (Quinn et al., 2023b); (Stafinski et al., 2022); (Pereira et al., 2021); (Goodman et al., 2019); (Lorente et al., 2019); (Mundy et al., 2019); (Bouvy et al., 2018); (Goncalves et al., 2018); (Maskineh and Nasser, 2018); (Tuffaha and Scuffham, 2018); (Nazareth et al., 2017); (Drummond, 2015); (Garrison et al., 2015); (Lu et al., 2015); (Navarria et al., 2015); (Klemp et al., 2011); (Hutton et al., 2007)
		Applicability and practicality	**(8)** (Al-Omar et al., 2021); (Quinn et al., 2023b); (Eichler et al., 2022); (Goble et al., 2017); (Seeley and Kesselheim, 2017); (Faulkner et al., 2016); (Annemans et al., 2011); (Neumann et al., 2011)
Performance metrics	**(27)** (Al-Omar et al., 2021); (Bhuiyan et al., 2024); (Dayer et al., 2024); (Trigg et al., 2024); (McPhail and Bubela, 2023); (Cheung et al., 2022); (Eichler et al., 2022); (Sandhu et al., 2022); (Simoens et al., 2022); (Kannarkat et al., 2020); (Lopez et al., 2020); (Goodman et al., 2019); (Holleman et al., 2019); (Duhig et al., 2018); (Goncalves et al., 2018); (Maskineh and Nasser, 2018); (Goble et al., 2017); (Jorgensen and Kefalas, 2017); (Pauwels et al., 2017); (Seeley and Kesselheim, 2017); (Faulkner et al., 2016); (Thompson et al., 2016); (Drummond, 2015); (Garrison et al., 2015); (Lu et al., 2015); (Neumann et al., 2011); (Carlson et al., 2009)	Un-define and non-unified performance metrics	**(16)** (Bhuiyan et al., 2024); (Dayer et al., 2024); (McPhail and Bubela, 2023); (Eichler et al., 2022); (Simoens et al., 2022); (Kannarkat et al., 2020); (Holleman et al., 2019); (Goncalves et al., 2018); (Maskineh and Nasser, 2018); (Goble et al., 2017); (Jorgensen and Kefalas, 2017); (Pauwels et al., 2017); (Faulkner et al., 2016); (Thompson et al., 2016); (Garrison et al., 2015); (Carlson et al., 2009)
		Insufficient data	**(9)** (Al-Omar et al., 2021); (Bhuiyan et al., 2024); (Dayer et al., 2024); (Cheung et al., 2022); (Simoens et al., 2022); (Lopez et al., 2020); (Duhig et al., 2018); (Seeley and Kesselheim, 2017); (Lu et al., 2015)
		Measurement issue	**(6)** (Sandhu et al., 2022); (Goodman et al., 2019); (Goble et al., 2017); (Drummond, 2015); (Garrison et al., 2015); (Neumann et al., 2011)
​	​	Surrogate endpoints	**(5)** (Dayer et al., 2024); (Trigg et al., 2024); (Simoens et al., 2022); (Pauwels et al., 2017); (Seeley and Kesselheim, 2017)
		Planed clinical outcomes	**(4)** (Al-Omar et al., 2021); (Dayer et al., 2024); (Duhig et al., 2018); (Seeley and Kesselheim, 2017)
Financial burden	**(27)** (Al-Omar et al., 2021); (Klemp et al., 2011); (Bhuiyan et al., 2024); (Hospod et al., 2024); (McPhail and Bubela, 2023); (Quinn et al., 2023a); (Kannarkat et al., 2020); (Mundy et al., 2019); (Bouvy et al., 2018); (Duhig et al., 2018); (Maskineh and Nasser, 2018); (Carlson et al., 2017); (Goble et al., 2017); (Nazareth et al., 2017); (Yeung et al., 2017); (Faulkner et al., 2016); (Thompson et al., 2016); (Garrison et al., 2015); (Lu et al., 2015); (Garrison et al., 2013); (Annemans et al., 2011); (Neumann et al., 2011); (Adamski et al., 2010); (Carlson et al., 2010); (Towse and Garrison, 2010); (Willis et al., 2010); (Carlson et al., 2009)	Implementation cost	**(19)** (Al-Omar et al., 2021); (Bhuiyan et al., 2024); (Hospod et al., 2024); (McPhail and Bubela, 2023); (Mundy et al., 2019); (Bouvy et al., 2018); (Duhig et al., 2018); (Maskineh and Nasser, 2018); (Carlson et al., 2017); (Goble et al., 2017); (Nazareth et al., 2017); (Yeung et al., 2017); (Thompson et al., 2016); (Garrison et al., 2015); (Lu et al., 2015); (Garrison et al., 2013); (Annemans et al., 2011); (Adamski et al., 2010); (Willis et al., 2010)
		Transaction cost	**(10)** (Al-Omar et al., 2021); (Quinn et al., 2023a); (Kannarkat et al., 2020); (Faulkner et al., 2016); (Lu et al., 2015); (Klemp et al., 2011); (Neumann et al., 2011); (Carlson et al., 2010); (Towse and Garrison, 2010); (Carlson et al., 2009)
Time consuming	**(20)** (Al-Omar et al., 2021); (Bartos, 2024); (Callenbach et al., 2024); (Dayer et al., 2024); (Trigg et al., 2024); (McPhail and Bubela, 2023); (Neumann et al., 2023); (Eichler et al., 2022); (Sandhu et al., 2022); (Goodman et al., 2019); (Triki et al., 2019); (Bouvy et al., 2018); (Faulkner et al., 2016); (Drummond, 2015); (Garrison et al., 2015); (Annemans et al., 2011); (Neumann et al., 2011); (Adamski et al., 2010); (Towse and Garrison, 2010); (Hutton et al., 2007)	Long time	**(11)** (Al-Omar et al., 2021); (Bartos, 2024); (Dayer et al., 2024); (Trigg et al., 2024); (McPhail and Bubela, 2023); (Sandhu et al., 2022); (Triki et al., 2019); (Bouvy et al., 2018); (Annemans et al., 2011); (Neumann et al., 2011); (Towse and Garrison, 2010)
		Delay access	**(7)** (Callenbach et al., 2024); (Eichler et al., 2022); (Goodman et al., 2019); (Faulkner et al., 2016); (Garrison et al., 2015); (Annemans et al., 2011); (Hutton et al., 2007)
		Longer follow-up	**(4)** (Neumann et al., 2023); (Bouvy et al., 2018); (Drummond, 2015); (Adamski et al., 2010)
Negotiation	**(16)** (Al-Omar et al., 2021); (Klemp et al., 2011); (McPhail and Bubela, 2023); (Sandhu et al., 2022); (Mundy et al., 2019); (Bouvy et al., 2018); (Duhig et al., 2018); (Goncalves et al., 2018); (Maskineh and Nasser, 2018); (Carlson et al., 2017); (Goble et al., 2017); (Nazareth et al., 2017); (Garrison et al., 2015); (Carlson et al., 2014); (Garrison et al., 2013); (Carlson et al., 2009)	Long and complex negotiations	**(9)** (Al-Omar et al., 2021); (McPhail and Bubela, 2023); (Mundy et al., 2019); (Goncalves et al., 2018); (Maskineh and Nasser, 2018); (Goble et al., 2017); (Nazareth et al., 2017); (Garrison et al., 2013); (Klemp et al., 2011)
		Resources needed for the negotiations	**(7)** (Sandhu et al., 2022); (Bouvy et al., 2018); (Duhig et al., 2018); (Carlson et al., 2017); (Garrison et al., 2015); (Carlson et al., 2014); (Carlson et al., 2009)
Privacy, confidentiality and trust	**(14)** (Al-Omar et al., 2021); (Klemp et al., 2011); (Bhuiyan et al., 2024); (Dayer et al., 2024); (Kim et al., 2023); (McPhail and Bubela, 2023); (Xoxi et al., 2022); (Kannarkat et al., 2020); (Mundy et al., 2019); (Bouvy et al., 2018); (Duhig et al., 2018); (Thompson et al., 2016); (Ferrario et al., 2015); (Carlson et al., 2009)	Privacy of patient data	**(6)** (Al-Omar et al., 2021); (Bhuiyan et al., 2024); (Dayer et al., 2024); (Kim et al., 2023); (McPhail and Bubela, 2023); (Duhig et al., 2018)
		Confidentiality issues	**(6)** (Al-Omar et al., 2021); (Bhuiyan et al., 2024); (Xoxi et al., 2022); (Mundy et al., 2019); (Ferrario et al., 2015); (Klemp et al., 2011)
		Trust issues between stakeholders	**(5)** (Al-Omar et al., 2021); (Kannarkat et al., 2020); (Bouvy et al., 2018); (Thompson et al., 2016); (Carlson et al., 2009)
Lack of expertise and knowledge	**(16)** (Al-Omar et al., 2021); (Bhuiyan et al., 2024); (McPhail and Bubela, 2023); (Neumann et al., 2023); (Xoxi et al., 2022); (Goodman et al., 2019); (Makad et al., 2019); (Bouvy et al., 2018); (Duhig et al., 2018); (Maskineh and Nasser, 2018); (Calabrese et al., 2017); (Clopes et al., 2017); (Faulkner et al., 2016); (Garrison et al., 2013); (Neumann et al., 2011); (Adamski et al., 2010)	Lack of expertise and knowledge	**(16)** (Al-Omar et al., 2021); (Bhuiyan et al., 2024); (McPhail and Bubela, 2023); (Neumann et al., 2023); (Xoxi et al., 2022); (Goodman et al., 2019); (Makad et al., 2019); (Bouvy et al., 2018); (Duhig et al., 2018); (Maskineh and Nasser, 2018); (Calabrese et al., 2017); (Clopes et al., 2017); (Faulkner et al., 2016); (Garrison et al., 2013); (Neumann et al., 2011); (Adamski et al., 2010)
Lack of guidelines	**(15)** (Al-Omar et al., 2021); (Bhuiyan et al., 2024); (Dayer et al., 2024); (Hospod et al., 2024); (McPhail and Bubela, 2023); (Bang and Lee, 2021); (Holleman et al., 2019); (Lorente et al., 2019); (Makad et al., 2019); (Yoo et al., 2019); (Bouvy et al., 2018); (Goncalves et al., 2018); (Goble et al., 2017); (Faulkner et al., 2016); (Lu et al., 2015)	Lack of clear operational guidelines	**(15)** (Al-Omar et al., 2021); (Bhuiyan et al., 2024); (Dayer et al., 2024); (Hospod et al., 2024); (McPhail and Bubela, 2023); (Bang and Lee, 2021); (Holleman et al., 2019); (Lorente et al., 2019); (Makad et al., 2019); (Yoo et al., 2019); (Bouvy et al., 2018); (Goncalves et al., 2018); (Goble et al., 2017); (Faulkner et al., 2016); (Lu et al., 2015)

(n), bold to reflect on the total number of identified references.

Regulatory framework challenge were the most frequently reported, referenced in 40 publications (41.6% of total publications). Within this category, “inefficient regulatory framework” constituted the primary concern (18 publications, 45.0% of regulatory barriers), followed by “regulations need to be updated/evolved” and “lack of exit strategy regulations” (10 publications each, 25.0%). These findings indicate fundamental structural limitations within existing regulatory and governance frameworks that hinder adaptive reimbursement decision-making.

Data resources and infrastructure barriers and challenges were identified in 39 publications (40.6%). “Data quality” concerns represented the most common sub-theme (17 publications, 43.6%), followed by “lack of data infrastructure” (14 publications, 35.9%) and “information system limitations” (13 publications, 33.3%). These findings underscore the central role of robust digital and data infrastructures for MEA execution, particularly for OBAs that rely on systematic outcome measurement and analysis.

Administrative burden was identified in 36 publications (37.5%), “high administrative burden” dominating this category (34 publications, 94.4%). The complexity inherent to MEAs, particularly OBAs, was itself cited as a barrier in 27 publications (28.1%). In addition, substantial “reporting and monitoring burden” was noted in 11 publications (30.5%), indicating the operational overhead associated with MEA management and its implications for healthcare system efficiency and financial sustainability.

Financial and economic challenges appeared in 27 publications (28.1%). “Implementation costs” constituted the most frequently cited concern (19 publications, 70.4%), followed by “transaction costs” (10 publications, 37.0%). These findings reflect persistent budget impact challenges within value-based reimbursement approaches and illustrate tensions between risk-sharing objectives and fiscal management.

Performance metrics challenges were also reported in 27 publications (28.1%), with “undefined and non-unified performance metrics “identified as the most significant issue (16 publications, 59.3%). This finding points to persistent methodological difficulties in establishing standardized outcome measures for MEA evaluation.

Overall, the barrier analysis demonstrates that successful MEA implementation requires addressing interconnected challenges across regulatory, data, administrative systems, and financial domains. The predominance of regulatory and data-related barriers suggests that foundational governance structures and technical capabilities represent the primary impediments to MEA adoption, while financial and administrative concerns present substantial operational hurdles to policy implementation.

## 4 Discussion

To our knowledge, this review represents the first systematic synthesis of global evidence on the core system-level components, governance, and operational requirements, across regulatory, policy, and governance dimensions, necessary for successful MEA policy design and implementation. The findings reveal a critical paradox within the MEA literature: although MEAs are widely recognized as valuable instruments for managing uncertainty, mitigating financial risk, and facilitating access to innovative therapies, the pathway to their effective design and operationalization remain poorly documented. For evolving or transitioning health systems, including Saudi Arabia’s, these limitations and gaps represent substantial operational and strategic risks. The existing literature provides insufficient documentation and guidance on system-level requirements, thereby constraining the capacity of policymakers and health system stewards to develop robust and implementable MEA policies.

For Saudi Arabia and similar systems in the region, the identified lacunae in global MEA policy literature represent both a challenge and an opportunity for innovation. Addressing implementation deficiencies and challenges observed in other health systems requires prioritizing investment in core system-level foundational components, rather than treating MEA adoption as an ancillary task. Overcoming entrenched regulatory hurdles demands a proactive legislative review and the creation of “regulatory sandboxes” to pilot innovative contracting mechanisms within a controlled environment. In parallel, aligning reimbursement approaches with innovative therapies such as ATMPs requires the development of tailored value assessment methodologies capable of capturing their full clinical and economic impact, supplemented by advanced risk stratification strategies to manage financial risk and evidentiary uncertainty. Given the absence of comprehensive operational guidance in the extant literature, structured policy learning, international collaboration, and the development of context-specific reimbursement models are essential for systems seeking sustainable pharmaceutical policy reform.

The findings further highlight a fundamental deficit in the literature concerning the core system components which are necessary to enable MEA adoption and operationalization. This “how-to” gap is particularly consequential for evolving or transitioning health systems. For a system like Saudi Arabia’s, developing such operational guidance constitutes a foundational requirement for successful MEA development, deployment and implementation.

Although situational analysis emerged as the most frequently addressed theme, appearing in 62.5% of publications, it largely focused predominantly on identifying barriers rather than outlining solutions. While the literature extensively documents operational challenges, including data infrastructure limitations, administrative burden, and monitoring complexity, it offers scant guidance on constructing the operational blueprints necessary to overcome them. The absence of standardized protocols for data collection, performance metric validation, and financial risk management under OBAs forces each system to devise its own processes, an approach that is inefficient and carries inherent risks. Although framework and implementation themes frequently co-occurred (51% of publications) the persistent lack of practical implementation tools leaves policymakers with a diagnosis but without a viable treatment plan.

Existing MEA frameworks and governance models remain fragmented and limited in scope and coherence; small-scale initiatives introduced in several countries in the early 2000s, followed by the European Commission’s 2011 survey on managed entry agreements in the European Union (Wenzl and Chapman, 2019). Most proposed frameworks share similar conceptual foundations, typically distinguishing between financial and non-financial agreements or combining elements of both. Despite growing policy attention, OBAs have rarely been executed as initially planned and have frequently evolved into discount-based arrangements following the analysis of the initial implementation experience (Dabbous et al., 2020). Although numerous frameworks have been reported, they tend to address isolated elements of MEA design, and a unified, comprehensive, and operationally detailed framework to support consistent national implementation remains lacking.

Across the literature, there is broad consensus that effective MEA governance frameworks should cover the full spectrum of conceptual, operational, and evaluative dimensions and incorporate principles of independence, data ownership, auditability, transparency, and appeal mechanisms (Klemp et al., 2011; MacLeod and Mitton, 2010). Given the inherently multidisciplinary nature of MEAs, effective implementation requires a cohesive and well-governed system that promotes synergistic stakeholder collaboration, with clearly defined roles, responsibilities, and decision-making authority.

The identification of an “inefficient regulatory framework” as the predominant governance barrier underscores a fundamental transition from conceptual feasibility to operational obstruction. The literature inadequately addresses how existing laws and regulations, largely designed for traditional, transactional purchasing models, actively constrain the conditional payments, data sharing, and multi-year financial commitments required by MEAs. More importantly, it provides insufficient prescriptive and actionable guidance required by policymakers and health systems in transition. This gap manifests in unresolved questions: which specific procurement laws require amendment to permit outcome-contingent payment?; which data governance provisions must be revised to enable secure real-world evidence generation while safeguarding patient confidentiality? How should budgetary rules be adapted to accommodate risk-sharing arrangements? This lack of specificity is particularly problematic for OBAs, where legal constraints on rebates, confidential pricing, and payment conditions often render MEAs legally impermissible. Consequently, for evolving or transitioning health systems, a paradigm shift is needed—reconceptualizing MEA development from isolated payment instruments (particularly FBAs) to integrated, cross-cutting policy strategies in which legislative modernization is not a theoretical exercise but a foundational prerequisite for creating a legally enabling environment.

### 4.1 Study limitations and strengths

As with any research, this systematic review is subject to some limitations that warrant acknowledgment. Restriction to English-language publications may have excluded relevant evidence from non-English-speaking jurisdictions. Furthermore, the rapid evolution of the MEA landscape suggests that the most recent policy innovations may not be fully represented within the analyzed corpus. Additionally, the heterogeneity of the included studies precluded quantitative synthesis. Finally, the decision to include only peer-reviewed literature may have excluded valuable insights and practical guidance frequently disseminated through grey literature sources. Furthermore, because the terms of MEAs are confidential and therefore unpublished, important operational details and implementation experiences are likely absent from the public domain entirely. Future research should therefore consider systematically searching grey literature and engaging with key stakeholders through interviews or surveys to capture unpublished operational terms, challenges, and solutions. Such efforts would complement and extend the findings of the present review. Despite these limitations, the review demonstrates substantial scholarly rigor through its exhaustive multi-database search strategy, its dual analytical focus on thematic domains and identification of previously underrecognized operational and regulatory implementation deficits. By systematically mapping the MEA policy ecosystem, from conceptual foundations to practical impediments, this review provides a unique high-level evidence base review for the global MEAs literature, highlighting critical system-level implementation requirements and challenges within the complex domain of pharmaceutical policy and reimbursement decision-making.

## 5 Conclusion

This systematic review moves beyond cataloging the literature or re-affirming the conceptual acceptance of MEAs, advancing instead toward a precise diagnosis of the critical evidence deficit surrounding practical and actionable requirements for MEA design and operationalization. It delineates several domains of paramount importance as core components and requirements for policymakers, regulators, and health system stewards seeking to incorporate MEAs into national pharmaceutical policy arsenal. These domains include, but are not limited to, operational frameworks, legislation, regulation, governance, and policy architecture. For evolving or transitioning health systems, systematic understanding and evaluation of these prerequisites constitutes a strategic imperative rather than a secondary consideration. Sustainable access to pharmaceutical innovation in these contexts depends on progressing from identifying what is needed to actively developing how to achieve it. This progression necessitates detailed design and implementation toolkits, clear legislative roadmaps, and fit-for-purpose payment models—elements that this review demonstrates to be critically absent from the global discourse. By concentrating reform efforts on these underdeveloped domains, health systems can strategically circumvent the pitfalls of legacy approaches and position themselves as pioneers in value-based, sustainable pharmaceutical policy.

## Data Availability

The original contributions presented in the study are included in the article/Supplementary Material, further inquiries can be directed to the corresponding author.
