# Supplementary material for: Mapping the landscape of managed entry agreements: a systematic review of global frameworks, system-level components, and implementation challenges
Source: Front Pharmacol. 2026 Apr 20;17:1803870. doi: 10.3389/fphar.2026.1803870 (PMC13136184; doi:10.3389/fphar.2026.1803870)
Supplement: Supplementary file 1 [file Table1.docx]

Supplementary Material 1

**Search strategy**

(“Managed entry agreement*” OR MEA OR “outcome based agreement*” OR “outcome based MEA” OR “performance based agreement*” OR OBA OR PBA OR “performance based MEA” OR “value based purchasing” OR VBP OR “risk sharing agreement*” OR RSA OR “performance linked coverage” OR “Performance linked reimbursement” OR “coverage with evidence development” OR CED OR “coverage with evidence generation” OR “Conditional coverage with evidence development” OR “patient access scheme*” OR “risk sharing scheme*” OR “special pricing arrangement*” OR “managed access strateg*” OR “managed access agreement*” OR “managed access scheme*” OR “cost sharing” OR “budget caps” OR “price volume agreement*” OR convention OR “managed access fund” OR “Innovative Medicines Fund” OR “conditional treatment continuation” OR “innovative agreement*” OR “Value based pricing” OR “Price volume agreement*” OR rebates OR “Pay for outcome” OR “Risk mitigation strateg*”)

AND

(“Drug reimbursement” OR “Conditional reimbursement” OR “innovative reimbursement process*” OR “Payment by Result*” OR “Pay by result*” OR “Payment at Result*” OR “Pay for performance” OR “innovative payment model*” OR IPM OR “alternative payment model*” OR APM OR “reimbursement”)

AND

(Framework OR, recommendation* OR strateg* OR model* OR guid* OR principle* OR rule* OR condition* OR term* OR operation*)

AND

(requirement* OR legal OR legislation* OR infrastructure OR governance OR polic* OR law OR system* OR information* OR data OR contract* OR procedure* OR regime OR act OR regulation* OR tool* OR approach* OR protocol* OR plan* OR structure OR agreement* OR arrangement* OR aspect* OR mechanism*)

AND

(Change* OR Modification* OR amend* OR process* OR reform* OR adjustment* OR transformation*)

AND

(Gap OR Area* OR room OR need* OR lack OR challeng* OR obstacle* OR barrier* OR limitation* OR drawback* OR issue* OR point* OR concern* OR opportunit* OR fall* OR problem*)

AND

(“health technolog*” OR Pharmaceutical* OR “medical device*” OR “medicinal product*” OR product* OR medicine* OR treatment* OR drug* OR therap* OR “Medicinal Device*” OR innovation* OR “breakthrough innovation*” OR orphan OR “innovative technolog*” OR diagnostic* OR procedure* OR “medical technolog*” OR intervention* OR “highly specialized technolog*” OR “high value technolog*” OR “high impact technolog*” OR “disruptive technolog*” OR “promising drug*” OR novel* OR“Highly speciali*ed medicin*” OR “advanced therapy medicinal product* OR IVD*”

AND

(access OR affordabilit* OR availabilit* OR “access to medicine*” OR “access to drug*” OR “access to medical technolog*” OR “access to medical device*” OR “access to diagnostic*” OR accessibilit*)
