# Supplementary material for: Mapping the landscape of managed entry agreements: a systematic review of global frameworks, system-level components, and implementation challenges
Source: Front Pharmacol. 2026 Apr 20;17:1803870. doi: 10.3389/fphar.2026.1803870 (PMC13136184; doi:10.3389/fphar.2026.1803870)
Supplement: Supplementary file 2 [file Table2.docx]

Supplementary Material 2

# Overall risk assessment table for included studies using the Critical Appraisal Skills Programme (CASP) checklist

| **No.** | **Author (year)** | **Overall Risk of Bias Assessment** |
| --- | --- | --- |
| 1 | Bartos (2024) | Low |
| 2 | Bhuiyan Khan, Gemme (2024) | Low |
| 3 | Callenbach, Goettsch (2024) | Low |
| 4 | Dayer, Drummond (2024) | Low |
| 5 | Hospodkova, Karasek (2024) | Low |
| 6 | Towse and Fenwick (2024) | Low |
| 7 | Trigg, Barnish (2024) | Low |
| 8 | Kim, Godman (2023) | Low |
| 9 | McPhail and Bubela (2023) | Low |
| 10 | Meresz and Gaal (2023) | Low |
| 11 | Neumann, Crummer (2023) | Low |
| 12 | Quinn, Ciarametaro (2023) | Low |
| 13 | Quinn, Ciarametaro (2023) | Low |
| 14 | Cheung, Cameron (2022) | Low |
| 15 | Eichler, Trusheim (2022) | Low |
| 16 | Russo (2022) | Low |
| 17 | Sandhu, Heidenreich (2022) | Low |
| 18 | Simoens, De Groote (2022) | Low |
| 19 | Stafinski, Glennie (2022) | Low |
| 20 | Xoxi, Rumi (2022) | Low |
| 21 | Al-Omar, Alghannam (2021) | Low |
| 22 | Bang and Lee (2021) | Low |
| 23 | Kim, Cho (2021) | Low |
| 24 | Pereira, Alves (2021) | Low |
| 25 | Gamba, Pertile (2020) | Low |
| 26 | Kannarkat, Good (2020) | Low |
| 27 | Lopez, Daniel (2020) | Low |
| 28 | Goodman, Villarivera (2019) | Low |
| 29 | Holleman, Uyl-de Groot (2019) | Low |
| 30 | Lorente, Antonanzas (2019) | Low |
| 31 | Makady, van Acker (2019) | Low |
| 32 | Mundy, Trowman (2019) | Low |
| 33 | Triki, Ash (2019) | Low |
| 34 | Wenzl (2019) | Low |
| 35 | Yoo, Kim (2019) | Low |
| 36 | Bouvy, Sapede (2018) | Low |
| 37 | Brown, Sheer (2018) | Low |
| 38 | Duhig, Saha (2018) | Low |
| 39 | Dunlop, Staufer (2018) | Low |
| 40 | Goncalves, Santos (2018) | Low |
| 41 | Kefalas, Ali (2018) | Low |
| 42 | Mahjoub, Odegaard (2018) | Low |
| 43 | Maskineh and Nasser (2018) | Low |
| 44 | Robinson, Mihalopoulos (2018) | Low |
| 45 | Tuffaha and Scuffham (2018) | Low |
| 46 | Calabrese, Cooke (2017) | Low |
| 47 | Carlson, Chen (2017) | Low |
| 48 | Clopes, Gasol (2017) | Low |
| 49 | Goble, Ung (2017) | Low |
| 50 | Grimm, Strong (2017) | Low |
| 51 | Jorgensen and Kefalas (2017) | Low |
| 52 | Kanavos, Ferrario (2017) | Low |
| 53 | Kim, Kim (2017) | Low |
| 54 | Nazareth, Ko (2017) | Low |
| 55 | Pauwels, Huys (2017) | Low |
| 56 | Seeley and Kesselheim (2017) | Low |
| 57 | Toumi, Jaroslawski (2017) | Low |
| 58 | Yeung, Suh (2017) | Low |
| 59 | Claxton, Palmer (2016) | Low |
| 60 | Faulkner, Lee (2016) | Low |
| 61 | Thompson, Henshall (2016) | Low |
| 62 | Drummond (2015) | Low |
| 63 | Ferrario and Kanavos (2015) | Low |
| 64 | Garattini, Curto (2015) | Low |
| 65 | Garrison, Carlson (2015) | Low |
| 66 | Lewis, Kerridge (2015) | Low |
| 67 | Lu, Lupton (2015) | Low |
| 68 | Navarria, Drago (2015) | Low |
| 69 | Carlson, Gries (2014) | Low |
| 70 | Gibson and Lemmens (2014) | Low |
| 71 | Launois, Navarrete (2014) | Low |
| 72 | Eckermann and Willan (2013) | Low |
| 73 | Garrison, Towse (2013) | Low |
| 74 | Morel, Arickx (2013) | Low |
| 75 | Evans (2012) | Low |
| 76 | Walker, Sculpher (2012) | Low |
| 77 | Wonder, Backhouse (2012) | Low |
| 78 | Annemans, Cleemput (2011) | Low |
| 79 | Barros (2011) | Low |
| 80 | Garattini and Casadei (2011) | Low |
| 81 | Jaroslawski and Toumi (2011) | Low |
| 82 | Jaroslawski and Toumi (2011) | Low |
| 83 | Klemp, Fronsdal (2011) | Low |
| 84 | Menon, Stafinski (2011) | Low |
| 85 | Neumann, Chambers (2011) | Low |
| 86 | Adamski, Godman (2010) | Low |
| 87 | Carlson, Sullivan (2010) | Low |
| 88 | McCabe, Stafinski (2010) | Low |
| 89 | Menon, McCabe (2010) | Low |
| 90 | Towse and Garrison (2010) | Low |
| 91 | Trueman, Grainger (2010) | Low |
| 92 | Willis, Persson (2010) | Low |
| 93 | Carlson, Garrison (2009) | Low |
| 94 | Williamson (2009) | Low |
| 95 | Hutton, Trueman (2007) | Low |
| 96 | Segal and Whinston (2002) | Low |
