# Supplementary material for: Mapping the landscape of managed entry agreements: a systematic review of global frameworks, system-level components, and implementation challenges
Source: Front Pharmacol. 2026 Apr 20;17:1803870. doi: 10.3389/fphar.2026.1803870 (PMC13136184; doi:10.3389/fphar.2026.1803870)
Supplement: Supplementary file 3 [file Table3.docx]

Supplementary Material 3

# Confidence in findings from qualitative evidence syntheses

| **Review finding** | **Studies**  **Contributing**  **to the Review**  **Finding (ref no.)** | **Assessment of**  **Methodological**  **Limitations** | **Assessment of**  **Relevance** | **Assessment**  **of Coherence** | **Assessment of**  **Adequacy** | **Overall**  **Assessment of Confidence** | **Explanation of**  **Judgement** |
| --- | --- | --- | --- | --- | --- | --- | --- |
| Existing global MEA frameworks | 10; 15; 18; 20; 22; 23; 24; 26; 27; 28; 33; 34; 35; 36; 37; 38; 39; 42; 43; 44; 45; 46; 53; 54; 59; 63; 66; 67; 69; 74; 76; 77; 79; 80; 83; 88; 89; 90; 91; 92; 93; 94; 95; 96; 98; 99; 100; 102; 104;105; 106; 107; 108; 109; 111; 112 | Minor Methodological  Limitations | Minor concerns about relevance (studies of managed entry agreements from four continents: Europe, North America, Asia, and Oceania, in addition to multicontinental studies) | Minor concerns (The finding demonstrates high thematic coherence across the body of evidence) | Minor concerns (Evidence base reached theoretical sufficiency across all primary sources) | Moderate confidence | This finding was  graded as  moderate  confidence  because of minor  concerns regarding  methodological  limitations,  relevance,  coherence, and  adequacy. |
| Foundational governance and operational prerequisites | All studies | Minor Methodological  Limitations | Minor concerns | Minor concerns | Minor concerns | Moderate confidence | This finding was  graded as  moderate  confidence  because of minor  concerns regarding  methodological  limitations,  relevance,  coherence, and  adequacy. |
| Pivotal stakeholders involved | 15; 18; 20; 21; 22; 23; 24; 25; 26; 27; 28; 29; 30; 31; 32; 33; 34; 35; 36; 37; 38; 39; 40; 41; 42; 44; 45; 46; 47; 48; 49; 50; 51; 52; 53; 54; 55; 56; 57; 58; 60; 61; 62; 63; 64; 65; 66; 67; 68; 69; 70; 71; 72; 73; 74; 75; 77; 78; 79; 80; 81; 82; 83; 84; 85; 86; 87; 88; 90; 91; 94; 95; 97; 98; 99; 100; 101; 102; 103; 104; 105; 106; 107; 108; 109; 110; 111 | Minor Methodological  Limitations | Minor concerns | Minor concerns | Minor concerns | Moderate confidence | This finding was  graded as  moderate  confidence  because of minor  concerns regarding  methodological  limitations,  relevance,  coherence, and  adequacy. |
| Implementation challenges | 15; 18; 20; 21; 22; 23; 24; 26; 27; 28; 29; 30; 31; 32; 33; 34; 36; 37; 38; 39; 40; 42; 44; 45; 46; 47; 48; 49; 50; 51; 52; 53; 55; 57; 60; 61; 62; 63; 64; 65; 66; 68; 69; 70; 71; 72; 73; 74; 75; 77; 78; 79; 80; 82; 84; 85; 86; 87; 90; 95; 101; 102; 103; 105; 106; 108; 109; 111 | Minor Methodological  Limitations | Minor concerns | Minor concerns | Minor concerns | Moderate confidence | This finding was  graded as  moderate  confidence  because of minor  concerns regarding  methodological  limitations,  relevance,  coherence, and  adequacy. |
