# Supplementary material for: Mapping the landscape of managed entry agreements: a systematic review of global frameworks, system-level components, and implementation challenges
Source: Front Pharmacol. 2026 Apr 20;17:1803870. doi: 10.3389/fphar.2026.1803870 (PMC13136184; doi:10.3389/fphar.2026.1803870)
Supplement: Supplementary file 4 [file Table4.docx]

Supplementary Material 4

# Full list of excluded records with reasons for exclusion

| **No.** | **Author** | **Year** | **Title** | **Reason for exclusion** |
| --- | --- | --- | --- | --- |
| 1 | Ellwein et al., | 1995 | Strategic issues in preventing cataract blindness in developing countries | excluded by title screening |
| 2 | Bartman | 1996 | Women's Access to Appropriate Providers Within Managed Care: Implications for Quality of Primary Care | excluded by title screening |
| 3 | YOUNG et al., | 1998 | Overview of Graduate Medical Education Funding Streams, Policy Problems, and Options for Reform | excluded by title screening |
| 4 | Casey et al., | 1998 | United Health Care of North Carolina | excluded by title screening |
| 5 | Conviser et al., | 2000 | Medicaid Managed Care Reimbursement for HIV and Its Implications for Access to Care | excluded by title screening |
| 6 | BARNETT et al., | 2000 | The Cost-Effectiveness of Methadone Maintenance | excluded by title screening |
| 7 | Savoca et al., | 2000 | The Civilian Labor Market Experiences of Vietnam-Era Veterans: The Influence of Psychiatric Disorders | excluded by title screening |
| 8 | COUTURIER et al., | 2000 | Effects of financial incentives on medical practice: results from a systematic review of the literature and methodological issues | systematic review |
| 9 | Dewa et al., | 2001 | Using Financial Incentives to Promote Shared Mental Health Care | excluded by title screening |
| 10 | Herbst et al., | 2001 | Management strategies for palliative care: Promoting quality, growth and opportunity | excluded by title screening |
| 11 | Rosenbaum et al., | 2002 | Evaluation of Agreements Between Managed Care Organizations and Providers of Community-Based Mental Illness and Addiction Disorder Treatments | excluded by title screening |
| 12 | Gurwitz et al., | 2002 | Hospital Transfer of Patients with Acute Myocardial Infarction: The Effects of Age, Race, and Insurance Type | excluded by title screening |
| 13 | Skarbinski et al., | 2002 | The Burden of Out-of-Pocket Payments for Health Care in Tbilisi, Republic of Georgia | excluded by title screening |
| 14 | Regan | 2002 | Utilizing an Intranet in Disease Management | excluded by title screening |
| 15 | Charney et al., | 2003 | Depression and Bipolar Support Alliance Consensus Statement on the Unmet Needs in Diagnosis and Treatment of Mood Disorders in Late Life | excluded by title screening |
| 16 | Lando et al., | 2003 | The impact of financial incentives and a patient registry on preventive care quality: increasing provider adherence to evidence-based smoking cessation practice guidelines | excluded by title screening |
| 17 | Jaffe | 2004 | REWARDING RESULTS: IMPROVING THE QUALITY OF TREATMENT FOR PEOPLE WITH ALCOHOL AND DRUG PROBLEMS | excluded by title screening |
| 18 | Tangcharoensathien | 2004 | Knowledge-based changes to health systems: the Thai experience in policy development | excluded by title screening |
| 19 | COX et al., | 2004 | Health Plan Member Experience With Point-of-Service Prescription Step Therapy | excluded by title screening |
| 20 | Downinga et al., | 2004 | Hypothesis: selective phosphodiesterase-5 inhibition improves outcome in preeclampsia | excluded by title screening |
| 21 | Wodchis et al., | 2004 | Payer Incentives and Physical Rehabilitation Therapy for Nonelderly Institutional Long-Term Care Residents: Evidence From Michigan and Ontario | excluded by title screening |
| 22 | Donohue et al., | 2005 | Helping Medicare Patients Benefit From the New Medicare Drug Benefit: An Overview With Practice Tips | excluded by title screening |
| 23 | Wroth et al., | 2005 | Application of Quality-Improvement Methods in a Community Practice: The Sandhills Pediatrics Asthma Initiative | excluded by title screening |
| 24 | Cochrane et al., | 2005 | Randomised controlled trial of the cost-effectiveness of water-based therapy for lower limb osteoarthritis | excluded by title screening |
| 25 | Lu et al., | 2005 | Recent developments in targeting access to high cost medicines in Australia | other reason |
| 26 | Stock et al., | 2006 | Population-Based Disease Management in the German Statutory Health Insurance Implementation and Preliminary Results | excluded by title screening |
| 27 | Herrera et al., | 2007 | Regional Palliative Care Program in Extremadura: An Effective Public Health Care Model in a Sparsely Populated Region | excluded by title screening |
| 28 | Navaneethan et al., | 2007 | Referral to Nephrologists for Chronic Kidney Disease Care: Is Non-Diabetic Kidney Disease Ignored? | excluded by title screening |
| 29 | DePue et al., | 2008 | Medicare Part D: Selected Issues for Plan Sponsors, Pharmacists, and Beneficiaries in 2008 | excluded by title screening |
| 30 | Glickman et al., | 2008 | Evidence-Based Perspectives on Pay for Performance and Quality of Patient Care and Outcomes in Emergency Medicine | MEA not for pharma |
| 31 | Lamden et al., | 2008 | General practice factors and MMR vaccine uptake: structure, process and demography | excluded by title screening |
| 32 | Rettig et al., | 2008 | Chronic Kidney Disease in the United States: A Public Policy Imperative | excluded by title screening |
| 33 | Hynd et al., | 2008 | The impact of co-payment increases on dispensings of government-subsidised medicines in Australia | excluded by title screening |
| 34 | Campbell et al., | 2009 | Effects of Pay for Performance on the Quality of Primary Care in England | MEA not for pharma |
| 35 | Abernethy et al., | 2009 | HORIZON SCAN: TO WHAT EXTENT DO CHANGES IN THIRD-PARTY PAYMENT AFFECT CLINICAL TRIALS AND THE EVIDENCE BASE? | excluded by title screening |
| 36 | KirschenBaum et al., | 2009 | Specialty pharmacies and other restricted drug distribution systems: Financial and safety considerations for patients and health-system pharmacists | excluded by title screening |
| 37 | Parker et al., | 2009 | Conclusions, Consensus, and Directions for the Future | excluded by title screening |
| 38 | Robson et al., | 2009 | Randomised preference trial of medical versus surgical termination of pregnancy less than 14 weeks’ gestation (TOPS) | excluded by title screening |
| 39 | Teerawattananon et al., | 2009 | Historical development of health technology assessment in Thailand | excluded by title screening |
| 40 | Goeree et al., | 2009 | Health Technology Assessment and Primary Data Collection for Reducing Uncertainty in Decision Making | excluded by title screening |
| 41 | Imamura et al., | 2010 | Systematic review and economic modelling of the effectiveness and cost-effectiveness of non-surgical treatments for women with stress urinary incontinence | systematic review |
| 42 | Logan et al., | 2010 | Coordinated clinical and financial analysis as a powerful tool to influence vendor pricing | other reason |
| 43 | Johnson et al., | 2010 | NCCN Oncology Risk Evaluation and Mitigation Strategies White Paper: Recommendations for Stakeholders | other reason |
| 44 | Schäfer et al., | 2010 | The Netherlands: Health System Review | excluded by title screening |
| 45 | Kang et al., | 2010 | In Situ Monitoring of Health in Older Adults: Technologies and Issues | excluded by title screening |
| 46 | McCabe et al., | 2010 | Economic Considerations in the Provision of Treatments for Rare Diseases | excluded by title screening |
| 47 | Oortwijn et al., | 2010 | The role of health technology assessment on pharmaceutical reimbursement in selected middle-income countries | excluded by title screening |
| 48 | Healy et al., | 2010 | Hospital Economics of Primary Total Knee Arthroplasty at a Teaching Hospital | excluded by title screening |
| 49 | Espı´n et al., | 2010 | Esquemas innovadores de mejora del acceso al mercado de nuevas tecnologı´as: los acuerdos de riesgo compartido | not in english |
| 50 | Jacob et al., | 2010 | Engaging and Empowering Patients to Manage Their Type 2 Diabetes, Part II: Initiatives for Success | excluded by title screening |
| 51 | Harris et al., | 2011 | Adaptive e-learning to improve dietary behaviour: a systematic review and cost-effectiveness analysis | systematic review |
| 52 | Antoun et al., | 2011 | Post-Soviet Transition: Improving Health Services Delivery and Management | excluded by title screening |
| 53 | Stafinski et al., | 2011 | To Fund or Not to Fund Development of a Decision-Making Framework for the Coverage of New Health Technologies | MEA not for pharma |
| 54 | Lee et al., | 2011 | The effects of new pricing and copayment schemes for pharmaceuticals in South Korea | other reason |
| 55 | DePalma | 2011 | Evolution of quality and surgical risk assessment in the USA | excluded by title screening |
| 56 | Beck et al., | 2012 | Counting the Cost of Not Costing HIV Health Facilities Accurately Pay Now, or Pay More Later | excluded by title screening |
| 57 | Frame et al., | 2012 | Assessment of Risk Evaluation and Mitigation Strategies in Oncology: Summary of the Oncology Risk Evaluation and Mitigation Strategies Workshop | other reason |
| 58 | Faulkner et al., | 2012 | GBEMTI Perspectives: Value-Based Reimbursement for Medical Devices in the U.S. – Where Do We Stand? | MEA not for pharma |
| 59 | Fugel et al., | 2012 | Stratified medicine and reimbursement issues | excluded by title screening |
| 60 | Henschke et al., | 2012 | Structural changes in the German pharmaceutical market: Price setting mechanisms based on the early benefit evaluation | excluded by title screening |
| 61 | דן גרינברג | 2012 | הסכמי חלוקת סיכון - גישה חדשה באימוץ טכנולוגיות תרופתיות | not in English |
| 62 | KALOZOLTAN | 2012 | Kockazat-Megosztasi technikak alkalmazasa a kozepes jovedelmu orszagokban | not in English |
| 63 | Nicod et al., | 2012 | Commonalities and differences in HTA outcomes: A comparative analysis of five countries and implications for coverage decisions | excluded by title screening |
| 64 | Sambo et al., | 2013 | Health financing in the African Region: 2000–2009 data analysis | excluded by title screening |
| 65 | Glenton et al., | 2013 | Barriers and facilitators to the implementation of lay health worker programmes to improve access to maternal and child health: qualitative evidence synthesis (Protocol) | excluded by title screening |
| 66 | Bates et al., | 2013 | Recommendation by a law body to ban infant male circumcision has serious worldwide implications for pediatric practice and human rights | excluded by title screening |
| 67 | Chronaki et al., | 2013 | Remote monitoring costs, benefits, and reimbursement: a European perspective | excluded by title screening |
| 68 | Fischer et al., | 2013 | Transparency vs. closed-door policy: Do process characteristics have an impact on the outcomes of coverage decisions? A statistical analysis | excluded by title screening |
| 69 | Merlin et al., | 2013 | Assessing Personalized Medicines in Australia: A National Framework for Reviewing Codependent Technologies | excluded by title screening |
| 70 | Petersen et al., | 2013 | Effects of Individual Physician-Level and Practice-Level Financial Incentives on Hypertension Care: A Cluster Randomized Trial | MEA not for pharma |
| 71 | Simoens et al., | 2013 | Patient Co-payment and Adherence to Statins: A Review and Case Studies | excluded by title screening |
| 72 | Grover et al., | 2013 | Building A Health Care Workforce For The Future: More Physicians, Professional Reforms, And Technological Advances | MEA not for pharma |
| 73 | Lopert et al., | 2013 | Applying rapid ‘de-facto’ HTA in resource-limited settings: Experience from Romania | excluded by title screening |
| 74 | DAVIS | 2014 | Equal Treatment and Unequal Benefits: The Medicare Program | excluded by title screening |
| 75 | Gregory et al., | 2014 | Practical Perspectives on the Management of Overweight and Obesity | excluded by title screening |
| 76 | Burge et al., | 2014 | Diabetes and the Affordable Care Act | excluded by title screening |
| 77 | Drummond et al., | 2014 | A Comparative Analysis of Two Contrasting European Approaches for Rewarding the Value Added by Drugs for Cancer: England Versus France | excluded by title screening |
| 78 | Drummond et al., | 2014 | Orphan drugs policies: a suitable case for treatment | excluded by title screening |
| 79 | Jönsson et al., | 2014 | New cancer drugs in Sweden: Assessment, implementation and access | other reason |
| 80 | Radford et al., | 2014 | Factors Which Impact the Delivery of Genetic Risk Assessment Services Focused on Inherited Cancer Genomics: Expanding the Role and Reach of Certified Genetics Professionals | excluded by title screening |
| 81 | Huen et al., | 2014 | Smoking Cessation Is the Least Successful Outcome of Risk Factor Modification in Uninsured Patients with Symptomatic Peripheral Arterial Disease | excluded by title screening |
| 82 | Latham et al., | 2014 | Performance-Based Financial Incentives for Diabetes Care: An Effective Strategy? | MEA not for pharma |
| 83 | PRÉMONT et al., | 2014 | Trois types de stratégies des fabricants pour la fidélisation aux médicaments de marque | not in English |
| 84 | Tavianini et al., | 2014 | Up for the Challenge: Eliminating Peripherally Inserted Central Catheter Infections in a Complex Patient Population | excluded by title screening |
| 85 | Br¨ugger et al., | 2014 | DEVELOPMENT OF COVERAGE WITH EVIDENCE DEVELOPMENT FOR MEDICAL TECHNOLOGIES IN SWITZERLAND FROM 1996 TO 2012 | MEA not for pharma |
| 86 | Long et al., | 2014 | Evolving provider payment models and patient access to innovative medical technology | MEA not for pharma |
| 87 | Lee et al., | 2014 | Medical Device Reimbursement Coverage and Pricing Rules in Korea: Current Practice and Issues with Access to Innovation | MEA not for pharma |
| 88 | Rose et al., | 2015 | Intensive Comprehensive Aphasia Programs: An International Survey of Practice | excluded by title screening |
| 89 | Das et al., | 2015 | Exploring vaginal ring acceptability for contraception and sexually transmissible infection protection in India: a qualitative research study | excluded by title screening |
| 90 | Frank et al., | 2015 | Together 4 Health: Integrating Care for Vulnerable Populations | excluded by title screening |
| 91 | Gomez et al., | 2015 | Improving Maternal Care through a StateWide Health Insurance Program: A Cost and Cost-Effectiveness Study in Rural Nigeria | excluded by title screening |
| 92 | Gutierrez et al., | 2015 | Principles for consistent value assessment and sustainable funding of orphan drugs in Europe | excluded by title screening |
| 93 | McGuire et al., | 2015 | End of life or end of the road? Are rising cancer costs sustainable? Is it time to consider alternative incentive and funding schemes? | MEA not for pharma |
| 94 | Meara et al., | 2015 | Global Surgery 2030: evidence and solutions for achieving health, welfare, and economic development | excluded by title screening |
| 95 | Hoof et al., | 2015 | Cross-border reproductive care for law evasion: A qualitative study into the experiences and moral perspectives of French women who go to Belgium for treatment with donor sperm | excluded by title screening |
| 96 | Warren et al., | 2015 | A cross sectional comparison of postnatal care quality in facilities participating in a maternal health voucher program versus non-voucher facilities in Kenya | excluded by title screening |
| 97 | Wouters et al., | 2015 | Transitioning to a national health system in Cyprus: a stakeholder analysis of pharmaceutical policy reform | excluded by title screening |
| 98 | Foy | 2015 | Aligning Your Department with Administration for Smooth Sailing | excluded by title screening |
| 99 | Panteli et al., | 2015 | From market access to patient access: overview of evidence-based approaches for the reimbursement and pricing of pharmaceuticals in 36 European countries | excluded by title screening |
| 100 | Menon et al., | 2015 | Developing a Patient-Directed Policy Framework for Managing Orphan and Ultra-Orphan Drugs Throughout Their Lifecycle | excluded by title screening |
| 101 | Jorgenson et al., | 2015 | Reimbursement of licensed cell and gene therapies across the major European healthcare markets | excluded by title screening |
| 102 | Leon et al., | 2016 | Critical Thinking and Treatment Strategies for Wounds | excluded by title screening |
| 103 | Apte et al., | 2016 | Payment Reform: Unprecedented and evolving impact on Gynecologic Oncology | excluded by title screening |
| 104 | Awoonor-Williams et al., | 2016 | Does the operations of the National Health Insurance Scheme (NHIS) in Ghana align with the goals of Primary Health Care? Perspectives of key stakeholders in northern Ghana | excluded by title screening |
| 105 | Chee et al., | 2016 | Current State of Value-Based Purchasing Programs | excluded by title screening |
| 106 | Fonseca et al., | 2016 | CONTINUOUS GLUCOSE MONITORING: A CONSENSUS CONFERENCE OF THE AMERICAN ASSOCIATION OF CLINICAL ENDOCRINOLOGISTS AND AMERICAN COLLEGE OF ENDOCRINOLOGY | excluded by title screening |
| 107 | Kawalec et al., | 2016 | Relating Health Technology Assessment recommendations and reimbursement decisions in Poland in years 2012–2014, a retrospective analysis | excluded by title screening |
| 108 | Kesselheim et al., | 2016 | The High Cost of Prescription Drugs in the United States Origins and Prospects for Reform | excluded by title screening |
| 109 | McEnhill et al., | 2016 | Effect of Immigration Status on Outcomes in Pediatric Kidney Transplant Recipients | excluded by title screening |
| 110 | Thiart et al., | 2016 | Internet-Based Cognitive Behavioral Therapy for Insomnia: A Health Economic Evaluation | excluded by title screening |
| 111 | Oosterhoff et al., | 2016 | A Systematic Review of Health Economic Evaluations of Diagnostic Biomarkers | systematic review |
| 112 | Dragovich | 2017 | Advancing Value-Based Contracting | excluded by title screening |
| 113 | Manchikanti et al., | 2017 | Merit-Based Incentive Payment System: Meaningful Changes in the Final Rule Brings Cautious Optimism | excluded by title screening |
| 114 | Doshi et al., | 2017 | Reducing Out-of-Pocket Cost Barriers to Specialty Drug Use Under Medicare Part D: Addressing the Problem of “Too Much Too Soon” | excluded by title screening |
| 115 | Rosenbaum et al., | 2017 | Medicaid Payment and Delivery System Reform: Early Insights from 10 Medicaid Expansion States | excluded by title screening |
| 116 | Akhmetov et al., | 2017 | Innovative payer engagement strategies: will the convergence lead to better value creation in personalized medicine? | excluded by title screening |
| 117 | Kenworthy et al., | 2017 | Use of opioid substitution therapies in the treatment of opioid use disorder: results of a UK cost-effectiveness modelling study | excluded by title screening |
| 118 | King | 2017 | Health Care Efficiencies: Consolidation and Alternative Models vs. Health Care and Antitrust Regulation Irreconcilable Differences? | excluded by title screening |
| 119 | MONDAL et al., | 2017 | A REVIEW ON ENERGY EFFICIENT JOB SCHEDULING ALGORITHMS IN GREEN CLOUD COMPUTING | excluded by title screening |
| 120 | Suárez-Albela et al., | 2017 | A Practical Evaluation of a High-Security Energy-Efficient Gateway for IoT Fog Computing Applications | excluded by title screening |
| 121 | Tivey | 2017 | INAHTA IMPACT STORY: LEGISLATIVE AND ACCREDITATION REQUIREMENTS FOR OFFICE-BASED SURGERY IN AUSTRALIA | excluded by title screening |
| 122 | Utidjian et al., | 2017 | Pediatric asthma hospitalizations among urban minority children and the continuity of primary care | excluded by title screening |
| 123 | Wetering et al., | 2017 | The Challenge of Conditional Reimbursement: Stopping Reimbursement Can Be More Difficult Than Not Starting in the First Place! | excluded by title screening |
| 124 | Vijayasingham et al., | 2017 | Challenges for accessing and financing high-cost medicines in multipayer systems: case studies of multiple sclerosis in Malaysia | excluded by title screening |
| 125 | Watkins et al., | 2017 | Universal Health Coverage and Essential Packages of Care | excluded by title screening |
| 126 | Wiysonge et al., | 2017 | Financial arrangements for health systems in low-income countries: an overview of systematic reviews (Review) | systematic review |
| 127 | Albin | 2017 | Stratégies médicales à adopter face aux changements réglementaires concernant la prise en charge des médicaments du cancer (radiation ou non-inscription sur « la liste en sus ») | not in English |
| 128 | Degtiar | 2017 | A review of international coverage and pricing strategies for personalized medicine and orphan drugs | systematic review |
| 129 | Lee et al., | 2017 | Facilitators and Barriers to the Adoption of Pharmacogenetic Testing in an Inner-City Population | excluded by title screening |
| 130 | TARRICONE et al., | 2017 | KEY RECOMMENDATIONS FROM THE MEDTECHTA PROJECT | MEA not for pharma |
| 131 | Ferrario et al., | 2017 | The Implementation of Managed Entry Agreements in Central and Eastern Europe: Findings and Implications | theoretical frameworks |
| 132 | Habit et al., | 2018 | Appointment Reminders to Decrease 30-Day Readmission Rates to Inpatient Psychiatric Hospitals | excluded by title screening |
| 133 | Kamusheva et al., | 2018 | an Overview of the reimbursement Decision-Making Processes in Bulgaria as a reference country for the Middle-income european countries | excluded by title screening |
| 134 | Koenig et al., | 2018 | The Effects of Revision Total Hip Arthroplasty on Medicare Spending and Beneficiary Outcomes: Implications for the Comprehensive Care for Joint Replacement Model | excluded by title screening |
| 135 | Li et al., | 2018 | Recent Pricing Negotiations on Innovative Medicines Pilot in China: Experiences, Implications, and Suggestions | excluded by title screening |
| 136 | Nelissen et al., | 2018 | Pharmacy-based hypertension care employing mHealth in Lagos, Nigeria – a mixed methods feasibility study | excluded by title screening |
| 137 | Radu et al., | 2018 | Themed Section: Drug Policies in Central and Eastern Europe Drug Policy in Romania | excluded by title screening |
| 138 | Sklar et al., | 2018 | Medical Education and Health Care Delivery: A Call to Better Align Goals and Purposes | excluded by title screening |
| 139 | Spivack et al., | 2018 | No Permanent Fix: MACRA, MIPS, and the Politics of Physician Payment Reform | excluded by title screening |
| 140 | Thériault et al., | 2018 | Patterns of bronchial challenge testing in Canada | excluded by title screening |
| 141 | Xiong et al., | 2018 | Impact of universal medical insurance system on the accessibility of medical service supply and affordability of patients in China | MEA not for pharma |
| 142 | Villa et al., | 2018 | Accesso precoce al mercato: dalle approvazioni condizionate di EMA agli accordi negoziali particolari di AIFA | not in English |
| 143 | Wallerstedt et al., | 2018 | Balancing early access with uncertainties in evidence for drugs authorized by prospective case series – systematic review of reimbursement decisions | systematic review |
| 144 | Rachlis et al., | 2018 | The impact of drug coverage on viral suppression among people living with HIV in Ontario, Canada | excluded by title screening |
| 145 | Wilkins | 2019 | Designing Benefits and Payment Models for Innovative High-Investment Medications | excluded by title screening |
| 146 | Salek et al., | 2019 | Pan-Canadian Pharmaceutical Alliance (pCPA): Timelines Analysis and Policy Implications | excluded by title screening |
| 147 | Bilger et al., | 2019 | Using Adherence‑Contingent Rebates on Chronic Disease Treatment Costs to Promote Medication Adherence: Results from a Randomized Controlled Trial | excluded by title screening |
| 148 | Clare et al., | 2019 | Goal-oriented cognitive rehabilitation for early-stage Alzheimer’s and related dementias: the GREAT RCT | excluded by title screening |
| 149 | Heinonen et al., | 2019 | The transposition of the Patients’ Rights Directive in Finland—Difficulties encountered | excluded by title screening |
| 150 | Langley | 2019 | Another Rush to Judgment: The Imaginary Worlds of ICER and Recommendations in Duchenne Muscular Dystrophy | excluded by title screening |
| 151 | Lee et al., | 2019 | Innovation in Regulatory Science Is Meeting Evolution of Clinical Evidence Generation | excluded by title screening |
| 152 | Pearce et al., | 2019 | Health Technology Assessment and Its Use in Drug Policies: Singapore | excluded by title screening |
| 153 | Wang et al., | 2019 | Status of public–private partnership recognition and willingness to pay for private health care in China | excluded by title screening |
| 154 | Zerhouni et al., | 2019 | Impact of the Affordable Care Act on trauma and emergency general surgery: An Eastern Association for the Surgery of Trauma systematic review and meta-analysis | systematic review |
| 155 | Kim et al., | 2019 | Health Technology Assessment Challenges in Oncology: 20 Years of Value in Health | systematic review |
| 156 | Mathes et al., | 2019 | Pay for performance for hospitals (Review) | systematic review |
| 157 | Spinner et al., | 2019 | Regenerative Medicine and Cell Therapy in Orthopedics—Health Policy, Regulatory and Clinical Development, and Market Access | excluded by title screening |
| 158 | Walton et al., | 2019 | A Review of Issues Affecting the Efficiency of Decision Making in the NICE Single Technology Appraisal Process | excluded by title screening |
| 159 | Birnbaum et al., | 2019 | Versorgungsstrukturen und-konzepte für Menschenmit Varianten der Geschlechtsentwicklung | not in English |
| 160 | Jiana et al., | 2019 | Beijing's diagnosis-related group payment reform pilot: Impact on quality of acute myocardial infarction care | excluded by title screening |
| 161 | Maynoua et al., | 2019 | What is driving HTA decision-making? Evidence from cancer drug reimbursement decisions from 6 European countries | excluded by title screening |
| 162 | Lee | 2020 | ONTARIO HEALTH TECHNOLOGY ASSESSMENT SERIES Implantable Devices for Single-Sided Deafness and Conductive or Mixed Hearing Loss: A Health Technology Assessment | excluded by title screening |
| 163 | N’Dri et al., | 2020 | The Evolution of Disease State Management: Historical Milestones and Future Directions | excluded by title screening |
| 164 | Reilly, | 2020 | What’s Next for Specialty Medication Benefit Design and Reimbursement | excluded by title screening |
| 165 | Balderrama et al., | 2020 | When are Pharmaceuticals Priced Fairly? An Alternative Risk‑Sharing Model for Pharmaceutical Pricing | excluded by title screening |
| 166 | Dimitrovová et al., | 2020 | Effect of a national primary care reform on avoidable hospital admissions (2000–2015): A difference-in-difference analysis | excluded by title screening |
| 167 | McLaughlin et al., | 2020 | Pharmacare in Canada: The paediatric perspective | excluded by title screening |
| 168 | Nittas et al., | 2020 | Self-Monitoring App Preferences for Sun Protection: Discrete Choice Experiment Survey Analysis | excluded by title screening |
| 169 | Palm et al., | 2020 | An initiative to implement immediate postpartum long-acting reversible contraception in rural New Mexico | excluded by title screening |
| 170 | Radcliff et al., | 2020 | Cost-Effectiveness of Three Doses of a Behavioral Intervention to Prevent or Delay Type 2 Diabetes in Rural Areas | excluded by title screening |
| 171 | Shih et al., | 2020 | Reimbursement Lag of New Drugs Under Taiwan's National Health Insurance System Compared With United Kingdom, Canada, Australia, Japan, and South Korea | excluded by title screening |
| 172 | Vilendrer et al., | 2020 | An Incentive to Innovate: Improving Health Care Value and Restoring Physician Autonomy Through Physician-Directed Reinvestment | excluded by title screening |
| 173 | Walker et al.,1 | 2020 | Cost and cost-effectiveness of a simplified treatment model with direct-acting antivirals for chronic hepatitis C in Cambodia | excluded by title screening |
| 174 | Casula et al., | 2020 | The 2017 Italian reform on mandatory childhood vaccinations: Analysis of the policy process and early implementation | excluded by title screening |
| 175 | Омельяновский et al., | 2020 | Доступность генной терапии in vivo проблемы и решения | not in English |
| 176 | Abbas et al., | 2020 | Access to medicines - a systematic review of the literature | systematic review |
| 177 | Michelsen et al., | 2020 | Barriers and Opportunities for Implementation of Outcome-Based Spread Payments for High-Cost, One-Shot Curative Therapies | systematic review |
| 178 | Khan et al., | 2020 | Perceptions of Occupational Risk and Changes in Clinical Practice of United States Vitreoretinal Surgery Fellows during the COVID-19 Pandemic | excluded by title screening |
| 179 | Lawrence et al., | 2020 | Variability in skilled nursing facility screening and admission processes: Implications for value-based purchasing | excluded by title screening |
| 180 | Munasinghe et al., | 2020 | Referral patterns to primary mental health services in Western Sydney (Australia): an analysis of routinely collected data (2005– 2018) | excluded by title screening |
| 181 | Zenone et al., | 2020 | Crowdfunding abortion: an exploratory thematic analysis of fundraising for a stigmatized medical procedure | excluded by title screening |
| 182 | Ionov et al., | 2020 | Value-based approach to blood pressure telemonitoring and remote counseling in hypertensive patients | excluded by title screening |
| 183 | Dias et al., | 2020 | An integrative review of Managed Entry Agreements – chances and limitations | systematic review |
| 184 | Richard et al., | 2021 | Performance-based pharmacy payment models: key components and critical implementation considerations for successful uptake and integration | MEA not for pharma |
| 185 | Ronquest et al., | 2021 | The evolution of ICER’s review process for new medical interventions and a critical review of economic evaluations (2018-2019): how stakeholders can collaborate with ICER to improve the quality of evidence in ICER’s reports | excluded by title screening |
| 186 | Edgar et al., | 2021 | Overcoming barriers to biosimilar adoption: real-world perspectives from a national payer and provider initiative | excluded by title screening |
| 187 | Ayati et al., | 2021 | Pharmacogenomics Implementation and Hurdles to Overcome; In the Context of a Developing Country | excluded by title screening |
| 188 | Chao Ma et al., | 2021 | Does integrated medical insurance system alleviate the difficulty of using cross-region health Care for the Migrant Parents in China-- evidence from the China migrants dynamic survey | excluded by title screening |
| 189 | Nekui et al., | 2021 | Cost-Related Medication Nonadherence and its Risk Factors among Medicare Beneficiaries | excluded by title screening |
| 190 | Neves et al., | 2021 | Cost–Utility Analysis of Wide-Field Imaging as an Auxiliary Technology for Retinopathy of Prematurity Care in Brazil | excluded by title screening |
| 191 | O’Connell et al., | 2021 | Reimbursement systems as a barrier to sunscreen use for organ transplant recipients: A community pharmacist survey | excluded by title screening |
| 192 | Samanta et al., | 2021 | Underutilization of Epilepsy Surgery: Part II: Strategies to Overcome Barriers | excluded by title screening |
| 193 | Upadhyay et al., | 2021 | State abortion policies and Medicaid coverage of abortion are associated with pregnancy outcomes among individuals seeking abortion recruited using Google Ads: A national cohort study | excluded by title screening |
| 194 | Yang et al., | 2021 | An equity evaluation in stroke inpatients in regard to medical costs in China: a nationwide study | excluded by title screening |
| 195 | Patarnello et al., | 2021 | Nuovi criteri di definizione del prezzo di un farmaco in Italia: riflessioni e proposte per supportare valore ed innovazione | not in English |
| 196 | Collacott et al., | 2021 | A Systematic Review of Discrete Choice Experiments in Oncology Treatments | systematic review |
| 197 | Qiu et al., | 2021 | Challenges in the market access of regenerative medicines, and implications for manufacturers and decision-makers: a systematic review | systematic review |
| 198 | Blonda et al., | 2021 | How to Value Orphan Drugs? A Review of European Value Assessment Frameworks | excluded by title screening |
| 199 | Tarricone et al., | 2021 | Establishing a national HTA program for medical devices in Italy: Overhauling a fragmented system to ensure value and equal access to new medical technologies | MEA not for pharma |
| 200 | Lawlor et al., | 2021 | Accelerating patient access to oncology medicines with multiple indications in Europe | excluded by title screening |
| 201 | Voncina et al., | 2021 | Pricing and reimbursement of patent-protected medicines: challenges and lessons from South-Eastern Europe | excluded by title screening |
| 202 | Efthymiadou et al., | 2021 | Determinants of Managed Entry Agreements in the context of Health Technology Assessment: a comparative analysis of oncology therapies in four countries | theoretical frameworks |
| 203 | Facey et al., | 2021 | Implementing Outcomes‑Based Managed Entry Agreements for Rare Disease Treatments: Nusinersen and Tisagenlecleucel | based on data obtained from included studies within the systematic review |
| 204 | Wix et al., | 2022 | COVID-19 and the acceleration toward remote cancer care | excluded by title screening |
| 205 | Sherman et al., | 2022 | Specialty drug and health care utilization vary by wage level in employer-sponsored health plans | excluded by title screening |
| 206 | Kozik et al., | 2022 | Mobile health in preventive cardiology: current status and future perspective | excluded by title screening |
| 207 | Andrén et al., | 2022 | Therapist-Supported Internet-Delivered Exposure and Response Prevention for Children and Adolescents With Tourette Syndrome A Randomized Clinical Trial | excluded by title screening |
| 208 | Couchman et al., | 2022 | Cost-effectiveness of long-term intermittent catheterisation with hydrophilic and uncoated catheters in traumatic spinal cord injury in Australia | excluded by title screening |
| 209 | Bennis et al., | 2022 | Strategic Recommendations to Bridge the Gaps in Awareness, Diagnosis and Prevention of Heart Failure in the Middle East Region and Africa | excluded by title screening |
| 210 | Bian et al., | 2022 | Exploring challenges to nutrition intervention adherence using COM- B model among patients with wet agerelated macular degeneration: a qualitative study | excluded by title screening |
| 211 | Biswas et al., | 2022 | Cipher constrained encoding for constraint optimization in extended nucleic acid memory | excluded by title screening |
| 212 | Crable et al., | 2022 | Translating Medicaid policy into practice: policy implementation strategies from three US states’ experiences enhancing substance use disorder treatment | excluded by title screening |
| 213 | Doan et al., | 2022 | Caring for the whole person: transgender-competent HIV pre-exposure prophylaxis as part of integrated primary healthcare services in Vietnam | excluded by title screening |
| 214 | Gallegos et al., | 2022 | Improving Organizational Sustainability of an Urban Indian Health Clinic With an Innovative Pharmacy Model | excluded by title screening |
| 215 | Gensorowsky et al., | 2022 | Market access and value-based pricing of digital health applications in Germany | MEA not for pharma |
| 216 | Miller et al., | 2022 | Implementation of pharmacist-led HIV pre-exposure prophylaxis management to increase access to care at an academic internal medicine practice | excluded by title screening |
| 217 | Mohamed et al., | 2022 | Cost effectiveness of simplified HCV screening-and-treatment interventions for people who inject drugs in Dar-es-Salaam, Tanzania | excluded by title screening |
| 218 | Ngum et al., | 2022 | Evaluation of the Effectiveness and Efficiency of the East African Community Joint Assessment Procedure by Member Countries: The Way Forward | excluded by title screening |
| 219 | Normanno et al., | 2022 | Access and quality of biomarker testing for precision oncology in Europe | excluded by title screening |
| 220 | Parekh et al., | 2022 | Impact of Co-pay Assistance on Patient, Clinical, and Economic Outcomes | excluded by title screening |
| 221 | Pisana et al., | 2022 | Challenges and Opportunities With Routinely Collected Data on the Utilization of Cancer Medicines. Perspectives From Health Authority Personnel Across 18 European Countries | excluded by title screening |
| 222 | Sachs et al., | 2022 | Reforming Reimbursement for the US Food and Drug Administration’s Accelerated Approval Program to Support State Medicaid Programs | excluded by title screening |
| 223 | Scheijmans et al., | 2022 | The reimbursement for expensive medicines: stakeholder perspectives on the SMA medicine nusinersen and the Dutch Coverage Lock policy | other reason |
| 224 | Sistani et al., | 2022 | COVID-19 pandemic and telemental health policy reforms | excluded by title screening |
| 225 | Schans et al., | 2022 | A novel perspective on pharmaceutical R&D costs: opportunities for reductions | excluded by title screening |
| 226 | Kanagasabai et al., | 2022 | Interventions to improve access to care for abnormal uterine bleeding: A systematic scoping review | systematic review |
| 227 | Bonful et al., | 2022 | Developing a culturally tailored short message service (SMS) intervention for improving the uptake of cervical cancer screening among Ghanaian women in urban communities | excluded by title screening |
| 228 | Debie et al., | 2022 | Contributions and challenges of healthcare financing towards universal health coverage in Ethiopia: a narrative evidence synthesis | excluded by title screening |
| 229 | Edwards et al., | 2022 | The impact of the private sector co-payment mechanism (PSCM) on the private market for ACT in Nigeria: results of the 2018 cross-sectional outlet and household market surveys | excluded by title screening |
| 230 | Wu et al., | 2022 | The challenge of healthcare big data to China’s commercial health insurance industry: evaluation and recommendations | excluded by title screening |
| 231 | Xia et al., | 2022 | Interventions in hypertension: systematic review and meta-analysis of natural and quasi-experiments | systematic review |
| 232 | Zhuang et al., | 2022 | The Price-Quality Mismatch: Are Negotiated Prices for Total Joint Arthroplasty Associated With Hospital Quality in a Large California Health System? | excluded by title screening |
| 233 | Rim et al., | 2022 | Collaborating with the Centers for Disease Control and Prevention’s National Comprehensive Cancer Control Program to Increase Receipt of Ovarian Cancer Care from a Gynecologic Oncologist | excluded by title screening |
| 234 | Liu et al., | 2022 | Policy Updates on Access to and Affordability of Innovative Medicines in China | other reason |
| 235 | Shengnan et al., | 2022 | Using 5 consecutive years of NICE guidance to describe the characteristics and influencing factors on the economic evaluation of orphan oncology drugs | excluded by title screening |
| 236 | Efthymiadou et al., | 2022 | Impact of Managed Entry Agreements on availability of and timely access to medicines: an ex‑post evaluation of agreements implemented for oncology therapies in four countries | theoretical frameworks |
| 237 | Bayram et al., | 2023 | Oppression and internalized oppression as an emerging theme in accessing healthcare: findings from a qualitative study assessing first-language related barriers among the Kurds in Turkey | excluded by title screening |
| 238 | Ostan et al., | 2023 | End-of-life care for patients with cancer: Clinical, geographical, and sociocultural differences | excluded by title screening |
| 239 | Perdikouri et al., | 2023 | Cost and reimbursement analysis of end-of-life cancer inpatients. The case of the Greek public healthcare sector | excluded by title screening |
| 240 | Stoeckl et al., | 2023 | Assessing the Dynamics of the Mental Health Apple and Android App Marketplaces | excluded by title screening |
| 241 | Quinn et al., | 2023 | Value-based performance arrangements for chronic conditions: an economic simulation of Medicaid Drug Rebate Program reforms | MEA not for pharma |
| 242 | Bower et al., | 2023 | Diabetes prevention at scale: Narrative review of findings and lessons from the DIPLOMA evaluation of the NHS Diabetes Prevention Programme in England | excluded by title screening |
| 243 | Himmelfarb et al., | 2023 | Shared Decision-Making and Cardiovascular Health: A Scientific Statement From the American Heart Association | excluded by title screening |
| 244 | Groene et al., | 2023 | Covering digital health applications in the public insurance system: how to foster innovation in patient care while mitigating financial risks— evidence from Germany | MEA not for pharma |
| 245 | Hansen et al., | 2023 | Palliative care need screening and specialised referrals fell during the COVID- 19 pandemic: a nationwide register- based study | excluded by title screening |
| 246 | Jakovljevic et al., | 2023 | Successes and challenges of China’s health care reform: a four-decade perspective spanning 1985—2023 | excluded by title screening |
| 247 | Koski et al., | 2023 | Community pharmacist-provided test and treat programs for acute infectious conditions | excluded by title screening |
| 248 | Lentz et al., | 2023 | Overcoming Barriers to the Implementation of Integrated Musculoskeletal Pain Management Programs: A Multi-Stakeholder Qualitative Study | excluded by title screening |
| 249 | NYSTAD et al., | 2023 | National health registries – a ‘goldmine’ for studying noncommunicable disease occurrence in Norway – the NCDNOR project | excluded by title screening |
| 250 | Torres‑Rueda et al., | 2023 | Health Economics Research on Non‑surgical Biomedical HIV Prevention: Identifying Gaps and Proposing a Way Forward | excluded by title screening |
| 251 | Trapani et al., | 2023 | Impact of Prior Authorization on Patient Access to Cancer Care | excluded by title screening |
| 252 | Tutuk et al., | 2023 | ASSESSMENT OF MEDICAL TECHNOLOGIES IN THE FORMATION OF GOVERNMENT PROGRAMS TO ASSIST PATIENTS WITH RARE METABOLIC DISEASES | MEA not for pharma |
| 253 | Wong et al., | 2023 | Using claims data to predict pre-operative BMI among bariatric surgery patients: development of the BMI Before Bariatric Surgery Scoring System (B3S3) | excluded by title screening |
| 254 | Nkangu et al., | 2023 | A systematic review of the effect of performance-based financing interventions on out-of-pocket expenses to improve access to, and the utilization of, maternal health services across health sectors in sub-Saharan Africa | systematic review |
| 255 | Decker et al., | 2023 | Novel approach to decision making for orphan drugs | other reason |
| 256 | Hogervorst et al., | 2023 | Uncertainty management in regulatory and health technology assessment decision-making on drugs: guidance of the HTAi-DIA Working Group | excluded by title screening |
| 257 | Butani, et al., | 2023 | Expanding access to high-cost medicines under the Universal Health Coverage scheme in Thailand: Review of current practices and recommendations | excluded by title screening |
| 258 | Griffiths et al., | 2023 | Demonstrating proof of concept for value-based agreements in Europe: two real-world cases | based on data obtained from included studies within the systematic review |
| 259 | Yeo et al., | 2024 | Understanding Patient Preferences Regarding the Important Determinants of Breast Cancer Treatment: A Narrative Scoping Review | excluded by title screening |
| 260 | Aryankhesal et al., | 2024 | Exploring the landscape of health technology assessment in Iran: perspectives from stakeholders on needs, demand and supply | MEA not for pharma |
| 261 | Tuffuor et al., | 2024 | Inequities among patient placement in emergency department hallway treatment spaces | excluded by title screening |
| 262 | Hernandez et al., | 2024 | Cheaper is not always better: Drug shortages in the United States and a value-based solution to alleviate them | excluded by title screening |
| 263 | Oderda et al., | 2024 | Evolving oncology care management trends in the United States: A survey among health care decision makers | excluded by title screening |
| 264 | Ost et al., | 2024 | Economic Value of Bronchoscopy Technologies that Improves Sensitivity for Malignancy for Peripheral Pulmonary Lesions | excluded by title screening |
| 265 | Cannavale et al., | 2024 | A comparative analysis of pricing and reimbursement systems between Italy and Bulgaria | other reason |
| 266 | Andrén et al., | 2024 | Internet-Delivered Exposure and Response Prevention for Pediatric Tourette Syndrome 12-Month Follow-Up of a Randomized Clinical Trial | excluded by title screening |
| 267 | Bowser et al., | 2024 | Payment-related barriers to medications for opioid use disorder: A critical review of the literature and real-world application | excluded by title screening |
| 268 | Chua et al., | 2024 | Association Between Cost Sharing and Naloxone Prescription Dispensing | excluded by title screening |
| 269 | Levaggi et al., | 2024 | Timely, Cheap, or Risk-Free? The Effect of Regulation on the Price and Availability of New Drugs | other reason |
| 270 | Liang et al., | 2024 | Cost Sharing for Oral Lenvatinib Among Commercially Insured Patients | other reason |
| 271 | Mansfield et al., | 2024 | The Hurdle of Access to Emerging Therapies and Potential Solutions in the Management of Dyslipidemias | other reason |
| 272 | Nkem et al., | 2024 | Economic exclusion and the health and wellbeing impacts of the oil industry in the Niger Delta region: a qualitative study of Ogoni experiences | excluded by title screening |
| 273 | Vogler et al., | 2024 | Pricing, Procurement and Reimbursement Policies for Incentivizing Market Entry of Novel Antibiotics and Diagnostics: Learnings from 10 Countries Globally | other reason |
| 274 | Wang et al., | 2024 | A comprehensive value-based method for new nuclear medical service pricing: with case study of radium [223Ra] bone metastases treatment | excluded by title screening |
| 275 | Middendorf et al., | 2024 | Development, Pilot, and Evaluation of a Qualitative Documentation Tool for Pharmacists to Share High Impact Patient Intervention Stories | excluded by title screening |
| 276 | Ng et al., | 2024 | Comparative policy analysis of national rare disease funding policies in Australia, Singapore, South Korea, the United Kingdom and the United States: a scoping review | excluded by title screening |
| 277 | Nieto‐Gómez et al., | 2024 | Factors influencing the reimbursement of cancer drugs in Europe: A scoping review | other reason |
| 278 | Hain et al., | 2024 | The Transition of Care Between Emergency Department and Primary Care: An Integrative Systematic Review | systematic review |
| 279 | Ferreira-Gonzalez et al., | 2024 | Barriers and facilitators to next-generation sequencing use in United States oncology settings: a systematic review | systematic review |
| 280 | Wagenschieber et al., | 2024 | Impact of reimbursement systems on patient care – a systematic review of systematic reviews | systematic review |
| 281 | Greco et al., | 2025 | A Systematic Review of Challenges and Opportunities in the Implementation of Managed Entry Agreements for Advanced Therapy Medicinal Products | systematic review |
| 282 | Callenbach et al., | 2025 | Managed Entry Agreements for High‑Cost, One‑Off Potentially Curative Therapies: A Framework and Calculation Tool to Determine Their Suitability | theoretical frameworks |
